# Forecasting Intra-individual Changes of Affective States Taking into Account Inter-individual Differences Using Intensive Longitudinal Data from a University Student Dropout Study in Math

**DOI:** 10.1007/s11336-022-09858-6

**Published:** 2022-04-02

**Authors:** Augustin Kelava, Pascal Kilian, Judith Glaesser, Samuel Merk, Holger Brandt

**Affiliations:** 1 Methods Center, University of Tübingen, Tübingen, Germany; 2 Methods Center, University of Tuebingen, Tübingen, Germany; 3 Karlsruhe School of Education, Karlsruhe, Germany

**Keywords:** dynamic factor models, structural equation model, time series, forecasting, Bayesian, nonlinear

## Abstract

The longitudinal process that leads to university student dropout in STEM subjects can be described by referring to (a) inter-individual differences (e.g., cognitive abilities) as well as (b) intra-individual changes (e.g., affective states), (c) (unobserved) heterogeneity of trajectories, and d) time-dependent variables. Large dynamic latent variable model frameworks for intensive longitudinal data (ILD) have been proposed which are (partially) capable of simultaneously separating the complex data structures (e.g., DLCA; Asparouhov et al. in Struct Equ Model 24:257–269, 2017; DSEM; Asparouhov et al. in Struct Equ Model 25:359–388, 2018; NDLC-SEM, Kelava and Brandt in Struct Equ Model 26:509–528, 2019). From a methodological perspective, forecasting in dynamic frameworks allowing for real-time inferences on latent or observed variables based on ongoing data collection has not been an extensive research topic. From a practical perspective, there has been no empirical study on student dropout in math that integrates ILD, dynamic frameworks, and forecasting of critical states of the individuals allowing for real-time interventions. In this paper, we show how Bayesian forecasting of multivariate intra-individual variables and time-dependent class membership of individuals (affective states) can be performed in these dynamic frameworks using a Forward Filtering Backward Sampling method. To illustrate our approach, we use an empirical example where we apply the proposed forecasting method to ILD from a large university student dropout study in math with multivariate observations collected over 50 measurement occasions from multiple students ($N = 122$). More specifically, we forecast emotions and behavior related to dropout. This allows us to predict emerging critical dynamic states (e.g., critical stress levels or pre-decisional states) 8 weeks before the actual dropout occurs.

**Supplementary Information:**

The online version contains supplementary material available at 10.1007/s11336-022-09858-6.

## Introduction

Forecasting, explaining, and preventing university student dropout from science, technology, engineering, and mathematics (STEM) is an important issue both for economies and for individuals. In this paper, we introduce a new methodological approach which can be used to forecast critical states, allowing for real-time inferences on latent or observed variables based on ongoing data collection. After introducing the model and a Forward Filtering Backward Sampling (FFBS) method the forecasting approach relies on, we apply it to the substantive area of university student dropout in mathematics, illustrating the approach using an empirical example. In a brief simulation study, we examine the forecasting performance of the model. But first, we identify gaps in the existing student dropout literature and suggest that our new methodological approach based on intensive longitudinal data (ILD) is particularly well suited to studying potential contributing factors to university student dropout.

### University Student Dropout from STEM

STEM subjects (especially their first semester) have the highest rates of student dropout (e.g., Heublein, 2014; Heublein et al., 2017, for the German context; see also Burrus et al., 2013; Robbins et al., 2004; Witteveen & Attevell, 2017). This finding together with the impact of STEM subjects in modern economies makes the study of dropout in STEM particularly urgent. There is a large body of research on the topic, though various gaps remain which the current study aims to address. Two classic models of university dropout are Tinto’s model of student departure (Tinto, 1993) and, complementing it, Bean’s model of student attrition (Bean, 1980, 1983, 2005). Given the large number of factors which might be associated with dropout and dropout intentions, a choice of focus on either institutional/contextual or individual factors or the interaction of these has to be made by researchers. The present study focuses on individual factors which will be discussed in the next section.

### Attainment, Motivation, Affect: Main Effects and Interaction Effects

Not surprisingly, motivation has been shown to be involved in student dropout. Dresel and Grassinger (2013) show that both motivational state measured at the beginning of a course of study and changes in motivation over the course of the first semester have an effect on the intention to drop out or to change course. Ghassemi et al. (2017) frame university dropout as a goal attainment issue. They identified a link between motivational measures such as value expectancy and (psychological) cost measures on the one hand and psychological processes leading to university dropout on the other. Several meta-analyses also showed that non-cognitive factors such as motivational and emotional measures are related to the outcomes university success and retention (e.g., Richardson et al., 2012; Robbins et al., 2004).

Other studies suggest that the investigation of interaction effects ought to be considered. Dresel and Grassinger (2013) and Schnettler et al. (2020) include interaction effects in their studies, though with mixed results.

### A Process Perspective

Several authors discuss the procedural nature of the university dropout decision. Hence, longitudinal study designs are appropriate for studying it (e.g., Dresel & Grassinger, 2013; Ghassemi et al., 2017; Isphording & Wozny 2018; Schaeper, 2020; Schnettler et al., 2020; Witteveen & Attewell, 2017). However, the duration and frequency of data collection vary heavily between studies, ranging from simple pre–post designs to studies collecting ILD (Dietrich et al., 2017). For example, Witteveen and Attewell (2017) use longitudinal data to identify several latent states that are associated with patterns of course taking and show that graduation or nongraduation can be predicted based on a few semesters of transcript data.

The present study builds on this body of research, using an ILD approach in order to study university dropout and its precursor, the intention to drop out. Individual factors potentially linked to university dropout, the focus of the present study, include a wide variety of characteristics which can be either fairly stable over time and across situations (e.g., Kilian et al., 2020) or *changeable within an individual over time*. The latter are the reason for taking a longitudinal perspective, as outlined in this section. Both Dietrich et al. (2017) and Schnettler et al. (2020) stress the importance of investigating such *intra-individual changes* in addition to inter-individual differences since the latter may not capture individual psychological processes which have been shown to be linked to outcomes such as university dropout. Gender, cognitive ability and pre-university academic performance are examples of stable (inter-individual) characteristics, and characteristics. Motivational and affective states as well as goal orientation are examples of (intra-individual) states which can vary over time in response to external experiences and stimuli.

### Implications

Taken together, these findings point to the need for more research into possible interaction effects between factors contributing to university dropout. They also suggest that these factors may be located on two levels: One level comprises relatively stable personal characteristics or traits, and the other consists of situation specific psychological states. The investigation of a possible interaction between factors located on different levels appears particularly promising.

Given that factors on the state level can be volatile, they are best studied using a longitudinal design with high-frequency points of measurement given their changeable nature. Such a design would also make it possible to capture a third type of factor, time-dependent variables outside an individual’s control, i.e., external events which may influence (unobserved) heterogeneity of trajectories. This methodological challenge requires innovative techniques so that the theoretical conceptualization can be captured by appropriate statistical models. The next section outlines this paper’s aims arising from these issues.

### Aims of this Article

First, we describe the general setting of the empirical example, the sample properties, the data collection and measures that were administered. Second, we introduce briefly dynamic latent variable frameworks which address multivariate observations, unreliable measures, and separate influences of the different types of data levels (within-person and across-person variability as well as time-dependent information and heterogeneity). We explain the dynamic model that we analyze and how it corresponds to substantive research questions as outlined in the introductory section. Third, we describe the implementation of the Forward Filtering Backward Sampling (FFBS) procedure. We explain how the dynamic model can be used for forecasting (e.g., of critical states) and give information on so-called observation and evolution equations, priors, state, forecast distributions, updating and posterior probabilities which allow the prediction of states as well as the quantification of uncertainty. Fourth, in a small simulation study, we will examine the robustness of our procedure and address conditions that influence the stability of the empirical results produced by our forecasts. Finally, we will apply the forecasting procedure to the real data example. More specifically, we will forecast emotions and behavior (e.g., subjective experiences of overload, stress, positive and negative affective states, cognitive states) related to dropout. Furthermore, we will analyze different influences that were proposed by substantive research on several data levels (including cross-level interactions). We will show the correspondence between theoretical expectations and empirical results.

## Intensive Longitudinal Data from the SAM Study

### General Setting of the Study

The SAM (German acronym for university dropout in mathematics) study is a longitudinal study focusing on university dropout in mathematics students at a German university. It is well documented that, in Germany, approximately 40 percent of students drop out in the early phase of math studies (Heublein, 2014). This is considerably higher than the 33 percent dropout rate found at German universities on average across all subjects in Heublein’s study. Internationally, rates vary considerably by country, with the OECD average being around 31 percent (OECD, 2010). The majority of students dropout during their first semester. Therefore, large introductory courses in math, such as calculus and algebra, are suitable settings to examine dropout in this important STEM subject. In the SAM study, data were collected from a first semester cohort starting in the winter semester of 2017/2018 and attending a calculus lecture and accompanying tutorial sessions. Since the calculus syllabi are very similar across German universities, the cohort can be considered to be prototypical for the general phenomenon of student dropout from math in Germany.

### Sample Properties

Our sample consists of 122 students with an average (median) age of 19.60 (19) (SD = 1.49). Fifty-five (45.08%) students indicated to be female and 66 (54.09%) students to be male. The sample includes 14 students who major in mathematics B.Sc. (3 females), 57 teacher candidates in mathematics B.Ed. (39 females) and 44 in physics B.Sc. (9 females). A further 6 students were voluntary participants enrolled on other study programs. The sample mean for German GPA (Abitur) was 1.86 (SD = 0.53) (with 1 being the best grade and 4 the lowest pass grade), and the mean for the final math grade from school was 12.03 (SD = 2.30) (with 15 being the highest possible grade and 5 the lowest pass grade). No significant differences in these measures were found between female and male students.

### Data Collection and Measures

The data were derived from three sources. The first source was an initial assessment used to obtain information on the inter-individual level. Data were collected in particular on individual stable characteristics, such as scholastic performance and gender, during the second week of the first semester. Lecturers agreed to use a part of their lectures for students to complete the questionnaire. Only students who have not yet been admitted to the exam from a previous semester were considered. One hundred and eighty-two students were eligible to participate. Of the 154 students who gave their consent to the processing of the data, $N_1 = 122$ also voluntarily participated in the online surveys (see below), which we consider a high participation rate (122/182 = 67.03%). Furthermore, a collection of approved TIMSS items (e.g., Mullis et al., 2005) was used for the assessment of students’ a priori math performance. In order to obtain further information on students’ cognitive abilities (IQ), we used a German adaption of the Culture Fair Intelligence Test Scale 3 (CFT-3, Cattell & Weiß, 1980). In addition to these performance measures, information was available on students’ educational backgrounds such as high school performance, type of school attended and whether they had attended preparatory courses before university or an additional in-depth mathematics class at school. Socio-demographic measures included parental educational qualifications, whether students were from immigrant families, and their financial situation. Professional interests were measured using the AIST-R questionnaire which is based on Holland’s (1997) RIASEC model (Bergmann & Eder, 2005). We also obtained information on motivational aspects (Wigfield & Cambria, 2010), personality (Big Five personality traits—BFI-2-XS; Soto & John, 2017), and on positive (PAP) and negative affect (PAN; using the positive and negative affect schedule PANAS; Watson et al., 1988). Locus of control was measured through the IE-4 scale (Kovaleva et al., 2012).

As a second source, starting 1 week after the initial assessment, data on changeable individual affective and motivational states were collected to cover the level of potential intra-individual differences. This was carried out via a short (5 min) online survey three times per week. The average participation rate per assessment day was 49.34 (SD = 17.72) out of $N_1 = 122$ total students. On average, the students participated in 20.22 (SD = 15.47) out of 50 online surveys (i.e., $N_t=50$ measurement occasions over a period of 131 days). The items re-assessed students’ motivational states and the positive and negative affective states which had first been assessed via the initial questionnaire. In addition, data were collected on students’ current intention to quit their course, fear of failure, subjective feeling of being overwhelmed by the demands of the course, ability to follow the calculus course, assignments, and learning behavior.

As a third source, performance and attendance information for the accompanying tutorial sessions was collected once a week. One of the reasons for collecting data weekly rather than waiting to collate all this information at the end of the semester was to be able to obtain information on dropouts during the semester.

Participation in each part of the study was voluntary, and students’ written consent was obtained for the use of the information they had given for the purposes of the study and for matching the different data sources via anonymous codes.

## Model Specification

### Dynamic Factor and Structural Equation Models

In the past, dynamic factor analysis models were developed for the analysis of multivariate time series reflecting intra-individual changes on latent variables (e.g., Molenaar 1985; 2017; Zhang et al., 2008). Such models are time series models with a factor analytic structure. Over the past years, several extensions have been developed, for example, covering categorical variables in a Bayesian approach (e.g., Zhang & Nesselroade, 2007), hidden Markov models with missing data in both frequentist and Bayesian approaches (e.g., Asparouhov & Muthén, 2010; Hamaker & Grasman, 2012; Hamaker et al., 2016), or frequentist continuous-time models (e.g., Voelkle & Oud, 2013; Oud et al., 2018).

Frequentist multilevel extensions of dynamic factor analysis models have been published that separate intra-individual and inter-individual differences (e.g., Chow et al., 2011; Song & Zhang, 2014). Their Bayesian counterparts in continuous time (Oravecz et al., 2009, 2016; Lu et al., 2019) have also been proposed in the literature. In the Bayesian dynamic structural equation model framework (DSEM; Asparouhov et al., 2018), inter-individual differences are used to explain random effects on the within-level. However, unobserved heterogeneity (as time-varying latent classes) is not part of the model. In the Bayesian dynamic latent class analysis framework (DLCA; Asparouhov et al., 2017), it is possible to specify heterogeneous autoregressive structural equation models. Latent class membership follows a hidden Markov process which describes the unobserved heterogeneity. The transition probabilities depend on stable inter-individual differences. Intra-individual changes have class-specific patterns. Time-dependent information is not used to predict latent class membership (e.g., intention to quit).

Traditionally, in regime switching (RS) models, hidden Markov processes (with latent classes) are used to explain heterogeneous autoregressive relationships (e.g., Chow & Zhang, 2013; Dacco & Satchell, 1999; Dolan et al., 2005; Kim & Nelson, 1999; Hamaker & Grasman, 2012; Tadjuidje et al., 2009). The parameters on the intra-individual level vary depending on the class membership. In the context of RS models, nonlinear state-space processes (and a frequentist extended Kim filter estimation method) were proposed allowing for more complex relationships of variables within regimes (e.g., Chow & Zhang, 2013). Furthermore, Bayesian continuous time RS models were proposed (e.g., Lu et al., 2019), in which the regimes depend on inter-individual differences (latent variables and covariates). As an important extension, frequentist RS models were developed to include time-dependent covariates as predictors of the regimes (Tadjuidje et al., 2009). Observed variables on the intra-individual level drive the time-dependent individual-specific transition probabilities. Note that this feature will be important for our application to affective data, which are individual-specific and time-dependent. We will predict the transition probabilities with previous affective states of the individuals that change over time.

Unlike the previous models, we will include two additional features in the model of the transition probabilities: First, we will include latent (instead of observed) within-variables to explain the transition probabilities. Second, we will allow for nonlinearities of (inter-individual and intra-individual) latent variables in the transition probabilities model. Although nonlinear latent effects were already considered in previous work (e.g., Chow et al., 2011; Guo et al., 2012), a comprehensive ILD framework capable of taking into account all features (incl. multilevel modeling) had been missing. As a flexible Bayesian approach, the nonlinear dynamic latent class structural equation model framework (NDLC-SEM, Kelava & Brandt, 2019) combines the capabilities of the models mentioned above. It allows for the inclusion of time-dependent information (including intra-individual changes) for modeling unobserved time-dependent class membership and heterogeneity (e.g., unexpressed intentions to quit or affective states). Furthermore, stable inter-individual differences can also be used to explain changes between the unobserved time-dependent class memberships of individuals. On both the within and between level, nonlinear (semiparametric) effects can be specified and random effects are essential elements of the framework. In our analysis, we will use these key features of the comprehensive NDLC-SEM framework.

### The Specified Model for the Analysis of the ILD and the Forecasting

*Within-level measurement model* 17 observed variables were used to operationalize seven latent within-factors, which we interpret in terms of affective/cognitive states potentially associated with higher risk of dropout. All observed variables were centered using the mean from the first time point and inverted if necessary such that we expected higher values for persons with an intention to quit. We used the following state variables: Three observed variables measured the *subjective importance of the content* (i.e., attainment value in motivation theory; e.g., “content not important”) and two variables indicated how much of their time students felt they needed to invest in order to understand the content at the expense of other important activities (i.e., *cost* in motivation theory; e.g., “too much time”). Two observed variables were directly related to the *intention to quit* and measured thoughts about quitting or fears to fail the math course (e.g., “afraid to fail”). Two observed variables (e.g., “no understanding”) measured subjective assessment of students’ own *understanding*. Two observed variables (e.g., “*stress*”) measured how stressed students felt. Finally, three observed variables each measured the *positive* and *negative affective states* (“no PAP” and “PAN”). We assumed that the within-level measurement models were the same whether students intended to quit or not:

1 $$\begin{aligned} (\mathbf{Y }_{1it} | S_{it} = s)&= \varvec{\Lambda }_{10} \varvec{\eta }_{1its} + \varvec{\epsilon }_{1it}, \end{aligned}$$

($\mathbf{Y }_{1it} | S_{it} = s$) was the ($17 \times 1$) vector of the observed variables on the within level (indicated by the index 1) measured for an individual *i* ($i = 1 \ldots N$) at time point *t* ($t = 0 \ldots 50$) given that the individual *i* shows a discrete latent state $S_{it}$ at time point *t* which is either an intention to stay ($s=1$) or to quit ($s=2$). $\varvec{\eta }_{1its}$ was the ($7 \times 1$) vector of the seven normally distributed latent variables describing the continuous latent affective/cognitive states on the within level for an individual *i* at time point *t* in a discrete latent state *s* (with no intention to quit $s=1$ or the intention to quit $s=2$). $\varvec{\eta }_{1its}$ describes individual-specific system dynamics (e.g., intra-individual changes of affective states), which will be further explained below. $\varvec{\epsilon }_{1it}$ was the ($17 \times 1$) vector of normally distributed independent residual variables which were class-invariant, centered and with a time-independent variance of $\sigma _{\epsilon _{1j}}^2$ for each indicator *j* (with $j=1\ldots 17$).^1^ The residuals were uncorrelated with each other and the latent variables, both within and across time. There was no autocorrelation structure for them. Furthermore, the residuals were unexplained by the actual process. The ($17 \times 7$) factor loading matrix $\varvec{\Lambda }_{10}$ used a simple structure that represented the factor structure described above and standard identification constraints.

Note that if a manifest dropout for student *i* occurred at time point *t*, $S_{it}=\cdots =S_{iT}=2$ become observed values. Before that time point, $S_{it}$ is a discrete latent state (class) variable with missing values and no direct information is available. This ensures identification of the latent discrete state variable that is in line with the nomenclature “intention to quit” (see below).

$$\begin{aligned} {{S_{it}=}} {\left\{ \begin{array}{ll} 2, &{} \text {if person }i\text { has dropped out at time point }t, \\ \text {NA (with }s=1,2), &{} \text { otherwise. } \end{array}\right. } \end{aligned}$$

That is, $S_{it}$ is treated as partly observed where persons who have not dropped out have missing data (that will be imputed during estimation).

*Between-level measurement model* On the between level (indicated by the index 2), we specified one latent construct $\eta _{2i}$ (“students’ cognitive abilities”; IQ) based on three centered items on cognitive tasks (CFT-3) that were measured at baseline:

2 $$\begin{aligned} \mathbf{Y }_{2i}&= \varvec{\Lambda }_{2} \eta _{2i} + \varvec{\epsilon }_{2i}, \end{aligned}$$

with a ($3 \times 1$) factor loading matrix $\varvec{\Lambda }_{2}$, a normally distributed latent factor $\eta _{2i}\sim \mathrm {N}(0,\sigma _{\eta _{2}}^{2})$, and a ($3 \times 1$) vector $\varvec{\epsilon }_{2i}$ with centered, uncorrelated and normally distributed residual terms $\epsilon _{2ji}\sim \mathrm {N}(0,\sigma _{\epsilon _{2j}}^2)$ ($j=1\ldots 3$).

*Within-level structural model* We specified an AR(1) model for the seven continuous latent state variables using a class-specific nonlinear model that accounted for the hypothesis that cognitive pre-university measures moderate the motivational and self-regulatory aspects during university.^2^ We assumed that persons with an intention to quit generally have higher scores in the seven within-factors and that the relationships between the variables can change across the intention states (classes). The model can be described as follows:

3 $$\begin{aligned} (\varvec{\eta }_{1it} | S_{it} = s)&= \varvec{\alpha }_{1is} + \mathbf{B }_{1is} \varvec{\eta }_{1i,t-1} + \varvec{\zeta }_{1it}, \end{aligned}$$

($\varvec{\eta }_{1it} | S_{it} = s$) was the ($7 \times 1$) vector of the seven latent within-level variables of a person *i* at time point *t* given that this person *i* is in a discrete latent state $S_{it}=s$ at time point *t* (with $s=1,2$). $\varvec{\alpha }_{1is}$ was the ($7 \times 1$) vector of intercepts. $\mathbf{B }_{1is}$ was the ($7 \times 7$) diagonal matrix of autoregressive AR(1)-effects. We will describe $\varvec{\alpha }_{1is}$ and $\mathbf{B }_{1is}$ in detail below because they contain the between-level factor $\eta _{2i}$. $\varvec{\zeta }_{1it}$ was the ($7 \times 1$) vector of normally distributed, independent residual variables which were class-invariant, centered, and with a time-independent variance of $\zeta _{1jit}\sim \mathrm {N}(0,\sigma _{\zeta _{1j}}^2)$ for each latent within-variable *j* ($j=1\ldots 7$). The residuals were uncorrelated with each other and the latent variables, both within and across time.

*Between-level structural model* For the random intercept vector $\varvec{\alpha }_{1is}$ and the AR coefficient matrix $\mathbf{B }_{1is}$ specified on the within level, the following two models were used:

4 $$\begin{aligned} \varvec{\alpha }_{1is}&= \varvec{\alpha }_{21s} + \varvec{\beta }_{2s} \eta _{2i} + \varvec{\zeta }_{2i} \end{aligned}$$

5 $$\begin{aligned} \mathbf{B }_{1is}&= \mathbf{B }_{1s} + \varvec{\Omega }_{2s} \eta _{2i} \end{aligned}$$

where $\varvec{\alpha }_{21s}$ is a ($7 \times 1$) class-specific intercept vector and $\varvec{\beta }_{2s}$ is the ($7 \times 1$) class-specific vector specifying the main effects of IQ on the within-level variables. $\mathbf{B }_{1s}$ is the ($7 \times 7$) diagonal matrix of autoregressive coefficients and the ($7 \times 7$) diagonal matrix; $\varvec{\Omega }_{2}$ includes the cross-level interactions that indicate whether IQ moderates the within-level relationships. $\varvec{\zeta }_{2i}$ is the ($7 \times 1$) vector of centered and independent normally distributed residuals which include all remaining unexplained stable inter-individual differences in each latent within-variable *j* ($j=1\ldots 7$) with $\zeta _{2ji}\sim \mathrm {N}(0,\sigma _{\zeta _{2j}}^2)$.

*Markov switching model* The elements (i.e., probabilities) of the $2 \times 2$ transition matrix of the time-dependent latent discrete states were given as

6 $$\begin{aligned} P(S_{it} = 1| S_{i,t-1} = 1)&= \frac{\exp (\nu _{it11})}{\exp (\nu _{it11}) + 1} \end{aligned}$$

7 $$\begin{aligned} P(S_{it} = 2| S_{i,t-1} = 1)&= 1 - P(S_{it} = 1| S_{i,t-1} = 1) \end{aligned}$$

8 $$\begin{aligned} P(S_{it} = 1| S_{i,t-1} = 2)&= P_{12} \quad \forall i \in \{ 1,...,N_1 \} \end{aligned}$$

9 $$\begin{aligned} P(S_{it} = 2| S_{i,t-1} = 2)&= 1 - P_{12} \quad \forall i \in \{ 1,...,N_1 \} \end{aligned}$$

with the probability to stay in state $S_{it}=1$ at time point *t*, i.e., no intention to quit, with no intention to quit ($S_{i,t-1}=1$) at time point $t-1$ modeled via

10 $$\begin{aligned} \nu _{it11}&= \gamma _{1} + \gamma _{2} \eta _{2i} + \varvec{\gamma }_{3} \varvec{\eta }_{1i,t-1} + \varvec{\gamma }_{4} \varvec{\eta }_{1i,t-1}\eta _{2i} \end{aligned}$$

where $\gamma _{1}$ is an intercept, $\gamma _{2}$ includes the effect of the baseline covariate IQ ($\eta _{2i}$), the ($1 \times 7$) coefficient vector $\varvec{\gamma }_{3}$ includes the lagged effects of the within-level variables, and the ($1 \times 7$) coefficient vector $\varvec{\gamma }_{4}$ includes three interaction effects between IQ and the self-regulatory variables from the within level (too much time, afraid to fail, stress).

For the transition probability $P(S_{it} = 1| S_{i,t-1} = 2) = P_{12}$, we only assumed that the probability was identical for all individuals *i* and time points *t* as identical [see Eq. (8)]. This resulted in a single parameter $P_{12}$ and did not depend on person- or time-dependent information due to both substantive reasons and model simplicity. The (transition) probability to return to state $S_{it}=1$ at time point *t*, after an intention to quit $S_{i,t-1}=2$ at time point $t-1$, was modeled via a uniform prior $P_{12} \sim unif(.0, .1)$. This prior was used in order to be able to better interpret the states from a substantive point of view, that is, we assumed that persons would rather slowly return to an intention not to drop out, once they developed an intention (in line with the Rubicon model; Achtziger & Gollwitzer, 2018).

Finally, the discrete state variables were modeled using a binomial distribution:

11 $$\begin{aligned} S_{it}&\sim \mathrm{{Bin}}(P(S_{it} = s | S_{i,t-1} = s'))\quad s, s' =1,2 \end{aligned}$$

We assumed that $S_{i1}=1$ holds for all persons $i=1\ldots N_1$, which means that on the first day, all students had no intention to quit.

**Fig. 1:**
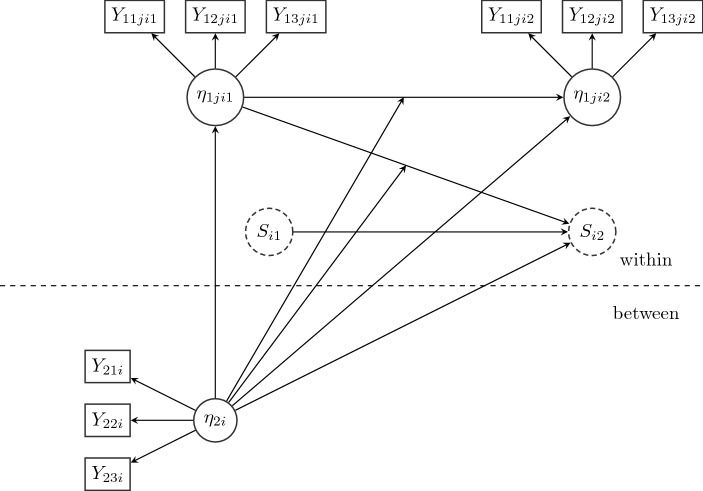
Path diagram for the final time series model for the *j*th latent within-factor $\eta _{1jit}$ (one out of seven) and the first two measurement occasions (out of 50). In the specified model, the latent within-factors have no cross-dependence. $S_{it}$ describes the latent discrete state variable (intention to quit; dashed circle). $\eta _{2i}$ shows the between-level factor with cognitive abilities.

In Fig. 1, the specified model is depicted for the *j*th latent within-factor at two time points. This within-factor $\eta _{1jit}$ has three indicators. The between-level latent factor ($\eta _{2i}$; IQ) is depicted in the lower part. The discrete latent state variable $S_{it}$ is shown as a dashed circle. For reasons of simplicity, the Markov switching model [see Eqs. (6)–(10)] is not depicted.

*Priors for the baseline model* The priors are summarized in the following equations^3^:

12 $$\begin{aligned}&\lambda _{10j} \sim \mathrm {TN}(0,1,0,\infty ) , \, j=1\ldots 6 \end{aligned}$$

13 $$\begin{aligned}&\lambda _{2j} \sim \mathrm {TN}(0,1,0,\infty ) , \, j=1\ldots 2 \end{aligned}$$

14 $$\begin{aligned}&\alpha _{21j(s=1)}, \beta _{2j(s=1)}, \beta _{1j(s=1)}, \omega _{2j(s=1)} \sim \mathrm {N}(0,1) , \, j=1\ldots 7 \end{aligned}$$

15 $$\begin{aligned}&\Delta \alpha _{21j(s=2)} \sim \mathrm {TN}(0,1,0,\infty ) , \alpha _{21j(s=2)} = \alpha _{21j(s=1)}+\Delta \alpha _{21j(s=2)} , \, j=1\ldots 7 \end{aligned}$$

16 $$\begin{aligned}&\Delta \beta _{2j(s=2)} \sim \mathrm {N}(0,1) , \, \beta _{2j(s=2)} = \beta _{2j(s=1)}+\Delta \beta _{2j(s=2)} , \, j=1\ldots 7 \end{aligned}$$

17 $$\begin{aligned}&\Delta \beta _{1j(s=2)} \sim \mathrm {N}(0,1) , \, \beta _{1j(s=2)} = \beta _{1j(s=1)}+\Delta \beta _{1j(s=2)} , \, j=1\ldots 7 \end{aligned}$$

18 $$\begin{aligned}&\Delta \omega _{2j(s=2)} \sim \mathrm {N}(0,1) , \, \omega _{2j(s=2)} = \omega _{2j(s=1)} + \Delta \omega _{2j(s=2)} , \, j=1\ldots 7 \end{aligned}$$

19 $$\begin{aligned}&\gamma _{1}, \gamma _{2} \sim \mathrm {N}(0,1) \end{aligned}$$

20 $$\begin{aligned}&\gamma _{3j} \sim \mathrm {N}(0,1) , \, j=1\ldots 7 \end{aligned}$$

21 $$\begin{aligned}&\gamma _{4j} \sim \mathrm {N}(0,1) , \, j=1\ldots 3 \end{aligned}$$

22 $$\begin{aligned}&\sigma _{\zeta _{1j}}^{-2}, \sigma _{\zeta _{2j}}^{-2} \sim \mathrm{{Gamma}}(9,4) , \, j=1\ldots 7 \end{aligned}$$

23 $$\begin{aligned}&\sigma _{\eta _{2}}^{-2} \sim \mathrm{{Gamma}}(9,4) \end{aligned}$$

24 $$\begin{aligned}&\sigma _{\epsilon _{1j}}^{-2} \sim \mathrm{{Gamma}}(9,4) , \, j=1\ldots 17 \end{aligned}$$

25 $$\begin{aligned}&\sigma _{\epsilon _{2j}}^{-2} \sim \mathrm{{Gamma}}(9,4) , \, j=1\ldots 3 \end{aligned}$$

where $\mathrm{{Gamma}}(a,b)$ is the Gamma distribution with hyperparameters *a*, *b*. TN(0,1,0,$\infty$) is a truncated normal distribution with mean equal to 0, variance equal to 1, and truncation parameters 0 and $\infty$. Note that $\Delta \alpha _{21j(s=2)}$ is a censored distribution, that is, we are assuming that persons who switch to $s=2$ (intention to quit) have higher values in the overall level of the *j*th factor. All items were inverted if necessary to ensure a straightforward interpretation.

*Identification* The identification of the model relates to two major aspects. First, the (continuous) latent variables (factors) need to be identified; this is achieved by using standard constraints for structural equation models (e.g., by use of a scaling item with factor loading fixed to one). Second, the latent discrete states need to be identified. This identification includes more aspects and is driven by actual differences in the observed response patterns. In addition, this identification is strongly related to the interpretation of the discrete state as “intention to drop out.” In order to achieve both, we follow a similar strategy as Jeon (2019). She describes how mixtures can be extracted using a more confirmatory method instead of traditional exploratory methods (such as Bauer, 2005). First, we use a censored distribution for the scale means such that persons in the state $s=2$ are constrained to have higher scores in all seven negative affect scales—which is in line with expectations about dropout. Second, we use these scales and theory-derived interactions with cognitive skills to predict the transition probabilities from one latent discrete state to the other. Third, we restrict the probability to return to an intention not to drop out, that is, we ensure that persons who have a high probability to quit do not change easily their mind at the following time points—again this is an assumption about the interpretation of the discrete state that is imposed using this confirmatory approach. By this conceptualization of the state “intention to drop out,” we give it a meaning that is close to crossing some kind of Rubicon (e.g., Heckhausen & Heckhausen, 2018). It is more than just playing with the idea of dropping out of the studies. And fourth, we use a partially observed latent class variable because information about persons who actually dropped out was available (see above).

*Implementation* The model was implemented in JAGS 4.2 (Plummer, 2017) and run via the R2jags package using automatic random stating values. Four chains each with 30,000 iterations were run with 25,000 iterations burn-in. Convergence was checked graphically via trace plots and density plots. The Rhat statistic was used to assess chain mixing, and a criterion of Rhat $<1.12$ was achieved for all parameters. Computation was conducted on a virtual machine of a server using 4 cores of an Intel XEON Gold 6244 3.6GHz CPU with 40GB RAM. Computation time for the empirical example was 23 hours. The computational burden for the simulation study was lower for each replication, as only three latent within-level factors were used and samples were smaller; for example, the condition with $N_1=50,N_t=25$ took about 50 minutes for each replication.

## Forecast Implementation

### Overview of the Procedure

Usually, when forecastings need to be performed in time series modeling, a standard way is to apply, for example, the frequentist Kalman (Kalman, 1960) or Kim filter (Kim & Nelson, 1999) as well as their further developments (e.g., Chow & Zhang, 2013) or their Bayesian counterparts (e.g., West & Harrison, 1997). Since the model that we propose is a hidden Markov model, we need a so-called forward–backward algorithm that also allows for inferences of the distribution of the hidden state variables (here: the intention to quit). As part of the algorithm, we predict latent variable scores and derive their distributions. Furthermore, past latent variable values are smoothed, given the occurrence of new observed values. Inferences on the latent variables build on one-step-back posterior distributions of the latent discrete variables.

The proposed *Forward Filtering Backward Sampling (FFBS)* algorithm is an adaptation of previous Bayesian forecasting work by West and Harrison (1997). The FFBS algorithm has parallels to both the extended Kim filter (EKF) and extended Kim smoother (EKS) (see Appendix in Chow & Zhang, 2013). Below, we will emphasize these parallels in the different steps of the FFBS algorithm and point out differences. However, the main improvement in the proposed FFBS algorithm is that it is capable of latent time-dependent information that is used in the parametrization of the transition probabilities (here: latent intra-individual changes of affective states). Individuals can switch their latent class membership (i.e., regime) in the time course depending on past time-varying latent variables. Given this model capability, the FFBS algorithm takes into account the potential class memberships to predict continuous latent variables and discrete latent states. As with the previous algorithms, the FFBS algorithm allows for forecasting and smoothing as well as for quantification of the uncertainty of the forecastings by providing forecasting distributions/intervals.

The FFBS algorithm that we describe here is fully integrated with the estimation using the Gibbs sampler in the JAGS software. In detail, the algorithm is implemented in a way, such that for each iteration $k = 1...K$ of the Gibbs sampler, which samples draws of the (conditional) distributions of the specified model parameters, samples of the different distributions belonging to the FFBS algorithm are drawn too (e.g., predictive density of a within-level latent factor $\eta _{1jit}$ at time *t* given the data up to time $t-1$).

Here, we forecast each of the *j*th within-level latent factor $\eta _{1jit}$ using the general framework described in chapters 14 and 16 in West and Harrison (1997) on multi-process (mixture) models and the general multivariate DLM, respectively. Following their description, we suggest a procedure that includes the following important aspects:

i.: Reformulate the model formulation from the above NDLC-SEM notation to observation and system equations as they are employed in West and Harrison (1997). The resulting models are state-specific quadruples of the form $\{{\mathbf {F}}_{jt},{\mathbf {G}}_{jts}, {\mathbf {V}}_{jts}, {\mathbf {W}}_{jts} \}$ that include information on individuals, parameters, observation and evolution error.
ii.: In order to account for the model evolving over time, four strata are defined for each pair of *t* and $t-1$. These relate to the two possible latent states of persons with or without an intention to drop out used in our model above (but could be extended for more states). These strata are denoted as $S_t=s$ and $S_{t-1}=s'$ with $s,s'=1,2$. They result in four possible combinations (e.g., $S_t=2,S_{t-1}=1$) which can potentially be observed for each person (e.g., if a person is classified as no intention to drop out at $t-1$ but with an intention to drop out at time point *t*). We will denote these strata as $(s,s')$.^4^
iii.: Within each stratum $(s,s')$, a within-level latent factor score for each person *i* is sampled (independent of the actual categorical class assignment). This results in the four forecast scores $(\eta _{1jit} | (s,s'), {\mathbf {D}}_{t-1})$.
iv.: Using the individual state probabilities $\pi _i(s,s')$ and $p_{i,t-1}(s')$ (defined below), the marginalized $\eta _{1jit}$ across the strata is received as the main outcome of the forecast.

In the following subsections, we use the notation “aspect i.”, “aspect ii.” etc. to refer to the labeled components of the algorithm. The subsections present the details of the FFBS algorithm, which are also summarized in the following pseudo code:
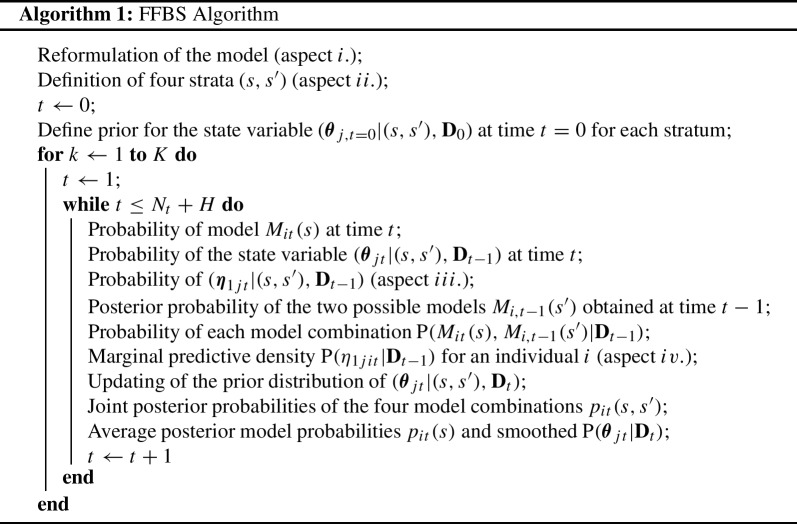


### Reformulation of the Model

In order to make use of the work on dynamic linear modeling (DLM) by West and Harrison (1997), we need to reformulate our model from above in terms of observation and system equations. This representation is equivalent to the model formulation from above within the NDLC-SEM framework (see supplementary online material). The observation equation relates the observed entity to latent continuous states; the system equation describes the evolution of these latent continuous states over time.

For aspect *i*. of Algorithm 1, we formulate within each state ($S_t=s$ with $s=1,2$) the state-specific $N_1$-dimensional latent factor $\varvec{\eta }_{1jts}$ as follows (cf. Equation 16.1 on p. 582 and p. 461 in West & Harrison, 1997):

26 $$\begin{aligned} \underbrace{\varvec{\eta }_{1jts}}_{N_1 \times 1}&= \underbrace{{\mathbf {F}}_{jt}}_{N_1 \times 5} \underbrace{\varvec{\theta }_{jts}}_{5\times 1}+ \underbrace{{\mathbf {v}}_{jts}}_{N_1 \times 1},\quad \mathbf{v}_{jts}\sim N( \underbrace{{\mathbf {0}}}_{N_1\times 1}, \underbrace{\mathbf{V }_{jts}}_{N_1\times N_1}) \end{aligned}$$

27 $$\begin{aligned} \underbrace{\varvec{\theta }_{jts}}_{5\times 1}&= \underbrace{{\mathbf {G}}_{jts}}_{5\times 5} \underbrace{\varvec{\theta }_{j,t-1,s}}_{5\times 1}+ \underbrace{{\mathbf {w}}_{jts}}_{5\times 1},\quad {\mathbf {w}}_{jts}\sim N( \underbrace{{\mathbf {0}}}_{5\times 1}, \underbrace{{\mathbf {W}}_{jts}}_{5\times 5}) \end{aligned}$$

with a ($N_1\times 5$) matrix ${\mathbf {F}}_{jt}=({\mathbf {1}}_{N_1}, \varvec{\eta }_{1j,t-1}, \varvec{\eta }_{2}, \varvec{\eta }_{1j,t-1} \cdot \varvec{\eta }_{2},\varvec{\zeta }_{2j})$, the ($N_1 \times N_1$) diagonal covariance matrix $\mathbf{V }_{jts} = \sigma ^2_{\zeta _{1j}}{\mathbf {I}}_{N_1\times N_1}$ of the so-called observational error ${\mathbf {v}}_{jts}$, a ($5 \times 5$) identity matrix ${\mathbf {G}}_{jts}={\mathbf {I}}_{5\times 5}$, and a ($5 \times 5$) covariance matrix ${\mathbf {W}}_{jts}={\mathbf {0}}_{5\times 5}$.

In general, the matrix ${\mathbf {F}}_{jt}$ includes all known values of independent variables; examples for the specification of ${\mathbf {F}}_{jt}$ (e.g., specific autoregressive models or regression models) can be found in West and Harrison (1997).

For the specified model, $\varvec{\theta }_{jts}$ is a five-dimensional system vector that includes the relevant time-invariant and class-specific regression coefficients $(\alpha _{21js}, \beta _{1jjs},\beta _{2js}, \omega _{2jjs}, 1)'$. Since our model does not include time-depending changes in the actual parameters (such as $\mathbf{B }_{1s}$), the errors ${\mathbf {w}}_{jts}$ (which is a five-dimensional vector) of the system equation are zero. Equation (27) implies that the parameter vector $\varvec{\theta }_{jts}$ does not change over time, i.e., $\varvec{\theta }_{jts}=\varvec{\theta }_{j,t-1,s}$. Note that in our empirical example these parts of the system equation become very simple/trivial. However, for other models this should not be the case. The FFBS algorithm presented below is more general.

Typically, the observation and system equations are summarized as a quadruple $\{{\mathbf {F}}_{jt},{\mathbf {G}}_{jts}, {\mathbf {V}}_{jts}, {\mathbf {W}}_{jts} \}$. The quadruple simplifies in our example further to $\{{\mathbf {F}}_{jt},{\mathbf {I}}_{5\times 5}, \sigma ^2_{\zeta _{1j}}{\mathbf {I}}_{N_1\times N_1}, {\mathbf {0}}_{5\times 5}\}$ (because these matrices do not change over time). For each person *i*, the state-specific model holds with probability $\pi _i(s,s')$ within each state (see the next subsection).

Note that in this formulation, random effects ($\varvec{\zeta }_{2j}$) are not marginalized out; instead, a person-specific estimate of the forecast is conducted using the sampled scores for $\varvec{\zeta }_{2j}$ for each person *i* from the posterior distribution. An alternative formulation that provides forecasts with marginalized random effects can be found in Gamerman and Migon (1993). The main difference between these two methods is that the formulation here allows us to forecast at an individual level as expressed with ($\varvec{\zeta }_{2j}$), whereas the formulation in Gamerman and Migon (1993) takes the variance of the random effects ($\sigma _{\zeta _{2j}}^{2}$) into account for the precision of the forecast intervals (across all persons *i*). Extensions to forecasts of the observed variables (i.e., the individual observed item responses $\mathbf{Y }_{1t}$) are straightforward and can be found, for example, in chapter 3.3.7 in Petris et al. (2009).

### Four Strata and Model Probability at Time *t*

For aspect *ii*. of Algorithm 1, we define the four strata $(s,s')$ as (1, 1), (1, 2), (2, 1), and (2, 2) corresponding to the four potential transitions form $t-1$ to *t*.

At time *t*, for the specified model for the *j*th latent factor, there are the following two potential models (cf. West & Harrison, 1997, Chapter 12) which correspond to the two latent discrete states (here: intention to quit vs. no intention to quit):

28 $$\begin{aligned} M_{it}(s):&\quad \{{\mathbf {F}}_{jt},{\mathbf {G}}, {\mathbf {V}}, {\mathbf {W}} \} \end{aligned}$$

We assume that for each time *t*, a model $M_{it}(s)$ applies with a probability to each person *i* (transition probability):

29 $$\begin{aligned} \pi _i(s,s')&= \mathrm {P}(M_{it}(s) | M_{i,t-1}(s') , {\mathbf {D}}_{t-1}) \end{aligned}$$

dependent on previous data ${\mathbf {D}}_{t-1}$ (and state $S_{i,t-1}=s'$ that individual *i* is in) at $t-1$. The data are given as a set of observed values of the within- and between-level observed indicators:

30 $$\begin{aligned} {\mathbf {D}}_{t-1}&:= \{\mathbf{Y }_{1|1..(t-1)}, \mathbf{Y }_{2}\} \quad \text {with} \end{aligned}$$

where $\mathbf{Y }_{1|1..(t-1)}$ is the $N_1 \times p \times (t-1)$ array of the within-level observations up to time $t-1$, and $\mathbf{Y }_{2}$ is the $N_1 \times q$ matrix of the between-level observations.

In other words, it is assumed that there is a model transition probability for each time point *t* given the data until $t-1$, namely ${\mathbf {D}}_{t-1}$. Note that in our model specification, we examine each latent within-factor *j* separately (due to computational reasons and reasons of simplicity). However, the FFBS algorithm is generalizable to other than AR structures that involve multiple within-factors.

### Prior for the State Variable at $t=0$

In order to start the FFBS algorithm/filter , a distribution of the continuous state variable is assumed. At time $t=0$, the prior for the state variable is given within each stratum $(s,s')$ as:

31 $$\begin{aligned} (\varvec{\theta }_{j,t=0} | (s,s'), {\mathbf {D}}_0)&\sim \mathrm {N}({\mathbf {m}}_{j0}(s,s'), {\mathbf {C}}_{j0}(s,s')) \end{aligned}$$

where the expectation ${\mathbf {m}}_{j0}(s,s')$ and covariance matrix ${\mathbf {C}}_{j0}(s,s')$ are priors within each stratum. For example, weakly informative priors can be chosen with a zero mean vector for ${\mathbf {m}}_{j0}(s,s')$ and a unit matrix for ${\mathbf {C}}_{j0}(s,s')$ (as in our example and simulation sections).

### Updating the Prior Distributions

When the $N_1$-dimensional vector $\varvec{\eta }_{1jt}$ (i.e., the *j*th within-level latent factor for all persons) is realized at time point *t* (i.e., via sampling from the posterior), the prior distributions are updated within each stratum $(s,s')$ (see p. 463 Equation (12.29) in West & Harrison, 1997).

32 $$\begin{aligned} (\varvec{\theta }_{jt} | (s,s'), {\mathbf {D}}_t)&\sim \mathrm {N}({\mathbf {m}}_{jt}(s,s'),{\mathbf {C}}_{jt}(s,s')) \end{aligned}$$

where ${\mathbf {m}}_{jt}(s,s')$ is a 5-dimensional vector and ${\mathbf {C}}_{jt}(s,s')$ is a ($5\times 5$)-dimensional matrix for time *t*, and each latent variable *j* under each of the regime combinations $(s,s')$. The distributional parameters are defined by

33 $$\begin{aligned} {\mathbf {m}}_{jt}(s,s')&= {\mathbf {a}}_{jt}(s,s') + \mathbf{A }_{jt}(s,s'){\mathbf {e}}_{jt}(s,s') \nonumber \\ {\mathbf {C}}_{jt}(s,s')&= {\mathbf {R}}_{jt}(s,s') - \mathbf{A }_{jt}(s,s') {\mathbf {Q}}_{jt}(s,s')\mathbf{A }_{jt}'(s,s') \nonumber \\ {\mathbf {e}}_{jt}(s,s')&=\varvec{\eta }_{1jt}-{\mathbf {f}}_{jt}(s,s') \nonumber \\ {\mathbf {A}}_{jt}(s,s')&={\mathbf {R}}_{jt}(s,s'){\mathbf {F}}'_{jt} {\mathbf {Q}}^{-1}_{jt}(s,s') \end{aligned}$$

where ${\mathbf {a}}_{jt}(s,s')$ is a five-dimensional vector, ${\mathbf {R}}_{jt}(s,s')$ is a ($5\times 5$)-dimensional matrix, ${\mathbf {Q}}_{jt}(s,s')$ is a ($N_1\times N_1$)-dimensional matrix, ${\mathbf {e}}_{jt}(s,s')$ and ${\mathbf {f}}_{jt}(s,s')$ are $N_1$-dimensional vectors. The remaining vectors and matrices are defined recursively as

34 $$\begin{aligned} {\mathbf {a}}_{jt}(s,s')&={\mathbf {G}} {\mathbf {m}}_{j,t-1}(s,s') \nonumber \\ {\mathbf {R}}_{jt}(s,s')&={\mathbf {G}}{\mathbf {C}}_{j,t-1}(s,s'){\mathbf {G}}'+{\mathbf {W}}\nonumber \\ {\mathbf {f}}_{jt}(s,s')&={\mathbf {F}}_{jt}{\mathbf {a}}_{jt}(s,s') \nonumber \\ {\mathbf {Q}}_{jt}(s,s')&={\mathbf {F}}_{jt}{\mathbf {R}}_{jt}(s,s'){\mathbf {F}}'_{jt} + {\mathbf {V}}. \end{aligned}$$

For $t=1$, we receive ${\mathbf {m}}_{j,t-1}(s,s'):=\mathbf{m}_{j0}(s,s')$ and ${\mathbf {C}}_{j,t-1}(s,s'):={\mathbf {C}}_{j0}(s,s')$ from the prior specification in Eq. (31).

Note that this step and the step with the derivation of joint posterior probabilities (see Sect. 4.11) correspond to the extended Kim smoother (EKS; see Chow & Zhang, 2013) where the latent continuous variable estimates and latent regime estimates are updated which are based on all information of the entire time series (up to this time point *t*). In other words, the prior distribution is updated such that $\varvec{\theta }_{jt}$ incorporates the information for all data that are realized so far.

### Probability of the State Variable at Time *t*

At time $t\le N_t$, the state variable $\varvec{\theta }_{jt}$ and the realized within-factor $\varvec{\eta }_{1jt}$ depend on the combinations of possible models at both $t-1$ and *t* in the four strata. Within each stratum, we have (see pp. 461–462 and Eqs. (12.25)–(12.27) in West & Harrison, 1997):

35 $$\begin{aligned} (\varvec{\theta }_{jt} | (s,s'), {\mathbf {D}}_{t-1})&\sim \mathrm {N}({\mathbf {a}}_{jt}(s,s'),{\mathbf {R}}_{jt}(s,s')) \end{aligned}$$

36 $$\begin{aligned} (\varvec{\eta }_{1jt} | (s,s'), {\mathbf {D}}_{t-1})&\sim \mathrm {N}({\mathbf {f}}_{jt}(s,s'),{\mathbf {Q}}_{jt}(s,s')) \end{aligned}$$

with ${\mathbf {a}}_{jt}(s,s'),{\mathbf {R}}_{jt}(s,s'),\mathbf{f}_{jt}(s,s'),{\mathbf {Q}}_{jt}(s,s')$ defined above. This part of the algorithm produces the smoothing part of $\varvec{\eta }_{1jt}$ when applying Eq. (44) to receive the marginalized factor scores.

Note that this step corresponds to parts A.1 and A.2 of the extended Kalman filter (EKF) in Chow and Zhang (2013). This step describes the probability of the latent continuous within-factor at time *t* given data until $t-1$ and four potential transitions between models (i.e., regime switches) from the latent discrete variable from $t-1$ to *t*. This is an important step, because it describes the four model combinations (regime switches/strata) and how these are related to the distribution of the continuous within-factor at time *t*.

### *H*-Steps-Ahead Forecast

For aspect iii. of Algorithm 1, the *H*-steps-ahead forecast distribution is given for all $t=N_t+1,\ldots , N_t+H$ within each stratum by (see p. 462, Eq (12.27)) and p. 584 in West & Harrison, 1997):

37 $$\begin{aligned} (\varvec{\eta }_{1jt}|(s,s'), {\mathbf {D}}_{t-1})&\sim N(\mathbf{f}_{jt}(s,s'),{\mathbf {Q}}_{jt}(s,s')) \end{aligned}$$

with

38 $$\begin{aligned} {\mathbf {f}}_{jt}(s,s')&= {\mathbf {F}}_{jt}{\mathbf {a}}_{jt}(s,s')\nonumber \\ {\mathbf {Q}}_{jt}(s,s')&= {\mathbf {F}}_{jt}{\mathbf {R}}_{jt}(s,s')\mathbf{F}'_{jt} + {\mathbf {V}} \end{aligned}$$

where we define in line with Eqs. (34) and (35) for $t=N_t+1$ (i.e., the one-step-ahead forecast)

39 $$\begin{aligned} {\mathbf {a}}_{j,N_t+1}(s,s')&= {\mathbf {G}}{\mathbf {m}}_{j,N_t}(s,s')\nonumber \\ {\mathbf {R}}_{j,N_t+1}(s,s')&= {\mathbf {G}}{\mathbf {C}}_{j,N_t}(s,s')\mathbf{G}'+{\mathbf {W}}. \end{aligned}$$

And for $t>N_t+1$, we define recursively

40 $$\begin{aligned} {\mathbf {a}}_{jt}(s,s')&= {\mathbf {G}}{\mathbf {a}}_{j,t-1}(s,s')\nonumber \\ {\mathbf {R}}_{jt}(s,s')&= {\mathbf {G}}{\mathbf {R}}_{j,t-1}(s,s'){\mathbf {G}}'+{\mathbf {W}}. \end{aligned}$$

This step describes for each of the four combinations of regime switches the predicted distribution of the *j*th latent within-variable $\varvec{\eta }_{1jt}$ within each stratum (in our example, an affective/cognitive state). This step is related to A.3 in the EKF as presented in Chow and Zhang (2013) which gives a one-step-ahead or *H*-steps-ahead prediction error. Note that A.3 in Chow and Zhang (2013) is related to the observed outcomes *Y*. The prediction error in the EKF is used to formulate the log-likelihood (for the parameter estimation). In the FFBS algorithm, we do not formulate a cost function based on the one-step-ahead prediction of the observed variable *Y*. Instead, we predict the continuous latent within-factor $\varvec{\eta }_{1jt}$ for each of the four model (regime) combinations.

### Posterior Probability of the Two Possible Models $M_{i,t-1}(s')$ Obtained at Time $t-1$

For each $s' = 1,2$ and individual *i*, the model $M_{i,t-1}(s')$ has a posterior probability $p_{i,t-1}(s')$ which is given at $t-1$ as

41 $$\begin{aligned} p_{i,t-1}(s')&= \mathrm {P}(M_{i,t-1}(s') | {\mathbf {D}}_{t-1}). \end{aligned}$$

Note that the notation $p_{i,t-1}(s')$ with the subscript ${t-1}$ of *p* implies that data are given until time $t-1$.

### Probability of Each Model Combination

Obtaining the forecast distribution of $\eta _{1jit}$ for an individual *i* unconditional on possible models involves mixing the (conditional) normal components utilizing their probabilities (see the next subsection). For each combination of ($s,s'$), the probabilities are given as

42 $$\begin{aligned} \mathrm {P}(M_{it}(s), M_{i,t-1}(s') | {\mathbf {D}}_{t-1})&= \mathrm {P}(M_{it}(s) | M_{i,t-1}(s'), {\mathbf {D}}_{t-1}) \, \mathrm {P}(M_{i,t-1}(s') | {\mathbf {D}}_{t-1}) \end{aligned}$$

43 $$\begin{aligned}&=\pi _i(s,s') p_{i,t-1}(s') \end{aligned}$$

with the definitions provided above in Eqs. (29) and (41).

This step is used to forecast the discrete latent time-dependent states (regimes; here: intention to quit) in the FFBS algorithm. Note that this step is also part of the Hamilton filter (see also Equation A.8 in Chow & Zhang, 2013). However, in contrast to the Hamilton filter, the prediction error decomposition function is not computed.

### Marginal Predictive Density

For aspect *iv*. of Algorithm 1, using the probabilities from Eqs. (37) and (43) from the previous steps, the marginal predictive density for $\eta _{1jit}$ for person *i* is the mixture of the four components:

44 $$\begin{aligned} \mathrm {P}(\eta _{1jit} | {\mathbf {D}}_{t-1})&= \sum _{s=1}^{2} \sum _{s'=1}^{2} \{ \pi _i(s,s') p_{i,t-1}(s') \mathrm {P}(\eta _{1jit} | (s,s'), {\mathbf {D}}_{t-1})\} \end{aligned}$$

Note that this step is used to forecast the continuous latent within-factor $\eta _{1jit}$ (e.g., an affective state of an individual *i*). It computes the distribution of the latent continuous within-factor $\eta _{1jit}$ given the data until $t-1$ (unconditional on the mixture components). This step corresponds to the collapsing process in the extended Kim filter (Kim & Nelson, 1999; Chow & Zhang, 2013). If no collapsing would be used, the four sets/components would lead to considerable computational burden. Here, the forecast of the continuous latent within-factor $\eta _{1jit}$ is unconditional of the potential regime switches [unlike Eq. (36)].

### Joint Posterior Probabilities

After realization of data ${\mathbf {D}}_{t}$, the model combination probabilities (for each individual *i*) are updated, such that data until time point *t* are incorporated. These joint posterior model probabilities given the data ${\mathbf {D}}_{t}$ across the four possible strata ($s,s'$) are given as [see Eqs. (37) and (43)]:

45 $$\begin{aligned} p_{it}(s,s')&= \mathrm {P}(M_{it}(s), M_{i,t-1}(s') | {\mathbf {D}}_{t}) \end{aligned}$$

46 $$\begin{aligned}&\propto \pi _i(s,s') p_{i,t-1}(s') \mathrm {P}(\eta _{1jit} | M_{it}(s), M_{i,t-1}(s'), {\mathbf {D}}_{t}) \end{aligned}$$

Note that the index *t* in $p_{it}(.)$ means that data are given up to time point *t*. The joint posterior model probabilities can be calculated by (see also p. 464 Eq. (12.30) in West & Harrison, 1997):

47 $$\begin{aligned} p_{it}(s,s')&= \frac{c_{jit} \pi _i(s,s') p_{i,t-1}(s')}{q_{jiit}(s,s')^{1/2}} \exp \{-.5 e_{jit}(s,s')^2 / q_{jiit}(s,s')\} \end{aligned}$$

where $q_{jiit}(s,s')$ is the *i*th element from the diagonal of ${\mathbf {Q}}_{jt}(s,s')$ (see Eq. (34)), and with the normalization constant $c_{jit}$ such that

$$\begin{aligned} \sum _{s=1}^{2} \sum _{s'=1}^{2} p_{it}(s,s') = 1 \end{aligned}$$

The normalization constant $c_{jit}$ is then

48 $$\begin{aligned} c_{jit}&= 1 / \sum _{s=1}^{2} \sum _{s'=1}^{2} \frac{\pi _i(s,s') p_{i,t-1}(s')}{q_{jiit}(s,s')^{1/2}} \exp \{-.5 e_{jit}(s,s')^2 / q_{jiit}(s,s')\} \end{aligned}$$

### Average Posterior Model Probabilities

In this final smoothing step, the last results are used to average the (conditional) posterior model probabilities [see Eq. (32)]. We obtain a smoothed distribution of the latent state variable (unconditional of the four components). The posterior model probabilities from above average using the four component mixtures, such that inferences about $\varvec{\theta }_{jt}$ are possible:

49 $$\begin{aligned} \mathrm {P}(\varvec{\theta }_{jt} | {\mathbf {D}}_t)&= \sum _{s=1}^{2} \sum _{s'=1}^{2} \mathrm {P}(\varvec{\theta }_{jt} | (s,s'), {\mathbf {D}}_{t}) p_{it}(s,s') \end{aligned}$$

These calculations complete the evolution and updating steps at time *t*.

Before moving to time $t+1$, it is important to free the joint posterior $p_{it}(s,s')$ from its dependence from potential models $s'$ at time $t-1$. Thus, we employ a collapsing procedure (over the potential models $s'$ at time $t-1$). We obtain

50 $$\begin{aligned} p_{it}(s) = \mathrm {P}(M_{it}(s) | {\mathbf {D}}_{t})&= \sum _{s'=1}^{2} p_{it}(s,s') \end{aligned}$$

51 $$\begin{aligned} \mathrm {P}(M_{i,t-1}(s') | {\mathbf {D}}_{t})&= \sum _{s=1}^{2} p_{it}(s,s') \end{aligned}$$

52 $$\begin{aligned} \mathrm {P}(M_{i,t-1}(s') | M_{it}(s), {\mathbf {D}}_{t})&= p_{it}(s,s') / p_{it}(s) \end{aligned}$$

Equation (50) is the model probability at time *t* (given data ${\mathbf {D}}_{t}$). Equation (51) is the smoothed one-step back posterior probability over potential models at time $t-1$. Equation (52) gives the posterior probability of the different models at $t-1$ (given data ${\mathbf {D}}_{t}$) conditional on the potential models at *t*.

Using these three equations, Eq. (49) can also be expressed as

53 $$\begin{aligned} \mathrm {P}(\varvec{\theta }_{jt} | {\mathbf {D}}_t)&= \sum _{s=1}^{2} \mathrm {P}(\varvec{\theta }_{jt} | M_{it}(s), {\mathbf {D}}_t) p_{it}(s) \quad \text {with}\nonumber \\ \mathrm {P}(\varvec{\theta }_{jt} | M_{it}(s), {\mathbf {D}}_t)&= \sum _{s'=1}^{2} \mathrm {P}(\varvec{\theta }_{jt} | M_{it}(s), M_{i,t-1}(s'), {\mathbf {D}}_t) p_{it}(s, s')/p_{it}(s) \end{aligned}$$

or as an approximation (see p. 465 Eq. (12.35) in West & Harrison, 1997):

54 $$\begin{aligned} (\varvec{\theta }_{jt} | M_{it}(s), {\mathbf {D}}_t)&\sim \mathrm {N}(\mathbf{m }_{jt}(s),\mathbf{C }_{jt}(s)) \quad \text {with}\nonumber \\ \mathbf{m }_{jt}(s)&= \sum _{s'=1}^{2} \mathbf{m }_{jt}(s,s') p_{it}(s,s')/p_{it}(s) \nonumber \\ \mathbf{C }_{jt}(s)&= \sum _{s'=1}^{2} \{ \mathbf{C }_{jt}(s,s') + (\mathbf{m }_{jt}(s) - \mathbf{m }_{jt}(s,s')) (\mathbf{m }_{jt}(s) - \mathbf{m }_{jt}(s,s'))'\} \nonumber \\&\quad \times p_{it}(s,s')/p_{it}(s) \end{aligned}$$

which collapses the posterior distribution to a single normal distribution for each potential model at time *t*. By this, the dependency on the joint posterior distribution is removed (compare Eq. (49)).

Note that this collapsing procedure and the simplification reduce computational time. In our implementation, we do not use this step, but provide it here. This step is also part of the EKS (see A.10 and A.11 in Chow & Zhang, 2013). Similar to the collapsing process in the EKF, in the smoother, the posterior model probabilities are averaged to obtain smoothed distributions of the continuous latent state variables (unconditional of the four components).

## Simulation Study

In this section, we present a small simulation study that evaluates the performance of the model specified within the NDLC-SEM framework for the purpose of forecasting based on the FFBS method as described above. We restrict our simulation study to the model specified in the example section below (abbreviated NDLC-SEM). We investigate how the forecast performance depends on sample size ($N_1$) and number of measurement occasions ($N_t$).

### Data Generation

Data were generated according to the model presented in Eqs. (1)–(11). In contrast to the empirical example, we used only three latent factors instead of seven (due to computational constraints). For each factor, three observed variables were generated. Population level parameters are based on the estimates obtained from the empirical example. We calculated means and standard deviations for each parameter across the seven within-scales we had analyzed in the empirical data (see Table 2 and Figure 4 in the Online Appendix). Parameters for each replication were randomly sampled from normal distributions using these population values. This allowed us to consider more general model specifications that include the different scales from the example but might be generalizable to other scales (that follow similar dynamic patterns).

Data were generated for $N_1=25$ versus $N_1=50$ (individuals; e.g., students) as well as $N_t=25$ versus $N_t=50$ measurement occasions. Data for additional $N_{t+}=10$ measurement occasions were generated and used to evaluate model performance after model estimation (see details below).

### Model Estimation and Evaluation

The NDLC-SEM and its forecast were implemented according to the description provided above with priors given in Eqs. (12,13,14,15,16,17,18,19,20,21,22,23,24)–(25). A forecast for the additional $N_{t+}=10$ time points after $N_t$ was conducted using the FFBS method. The NDLC-SEM was run with 2 chains and 10,000 iterations each with 5000 iterations burn-in. A total of $R=100$ data sets were generated under each condition. Convergence was checked for each replication using the Rhat statistic.

We used four major outcomes to evaluate the performance of the models. First, we investigated the *sensitivity and specificity* of the NDLC-SEM for the state extraction with regard to the actual discrete state membership generated for the data. We distinguished between overall measures, for the state estimation during the first $N_t$ measurement occasions, and the state forecast for the $h=1\ldots N_{t+}$ time points. Second, we calculated the average $95\%$ *coverage rates* for the factor scores based on the forecasting intervals for the forecast factor score estimates. Third, we used a *score function* $\delta _{h}$ to evaluate how well the forecast of the latent within factors replicated the actual factor scores of the three latent within factors by

55 $$\begin{aligned} \delta _h=\sum _i\sum _j \left( {\hat{\eta }}_{ijh}-\eta _{ijh}\right) ^2, \quad i=1\ldots N_1, j=1\ldots 3 \end{aligned}$$

for each forecast time point $h=1\ldots N_{t+}$ (cf. Gneiting, 2011). Finally, we investigated how precise the forecast was by calculating the *average width of the forecasting intervals* (FI) for the forecast factor score estimates at each forecast time point.

### Results

*State prediction* Table 1 shows the results for the state estimation and the state prediction. Sensitivity for the discrete state extraction was above 0.91 under all conditions. This indicated that persons who actually switched could reliably be detected. This also extended to the forecast of the discrete states. Specificity was lower with values between 0.83 and 0.88 for the time points in the interval 1 through $N_t$, that is, the prediction of the state membership for the observed data. Specificity was lower for the forecast time points with values ranging between 0.53 and 0.70; they were lower for smaller sample size $N_1=25$ compared to $N_1=50$. This was due to the fact that too many persons were forecast to switch to the latent state $s=2$ in this interval. It implied that in order to achieve acceptable specificity rates, at least $N_1=50$ individuals should be included. As a result of the chosen length of the chains with 10,000 iterations (due to computational time restrictions), it could be observed that for the small sample size $N_1=25$ an increasing time series from $N_t=25$ to $N_t=50$ lead to a lower specificity (0.62 vs. 0.53). Again, with a larger sample size $N_1=50$, this phenomenon could not be observed.

**Table 1:** Sensitivity and specificity for the latent class extraction using the NDLC-SEM and 95% coverage rates for the forecast intervals.

$N_t$	$N_1$	Sensitivity			Specificity			Coverage
		Overall	Observed data	Forecast	Overall	Observed data	Forecast	
25	25	0.92	0.91	0.93	0.83	0.85	0.62	0.90
	50	0.93	0.93	0.94	0.87	0.88	0.70	0.88
50	25	0.96	0.96	0.95	0.81	0.83	0.53	0.89
	50	0.94	0.95	0.94	0.85	0.86	0.70	0.88

Overall includes all time points, Observed data is restricted to the time points 1 through $N_t$, and Forecast is restricted to the forecast time points $h=1\ldots N_{t+}.$

*Coverage rates* The last column in Table 1 shows the 95% coverage rates for the forecast intervals averaged across time points, persons and the three latent factors for the NDLC-SEM. Across all conditions, coverage rates were between 88% and 90%. The NDLC-SEM thus showed somewhat lower coverage rates than the nominal 95% level. This finding can be attributed to the lower specificity described above, that is, persons were classified too often to switch to the discrete state $s=2$ (i.e., intention to quit).

**Fig. 2:**
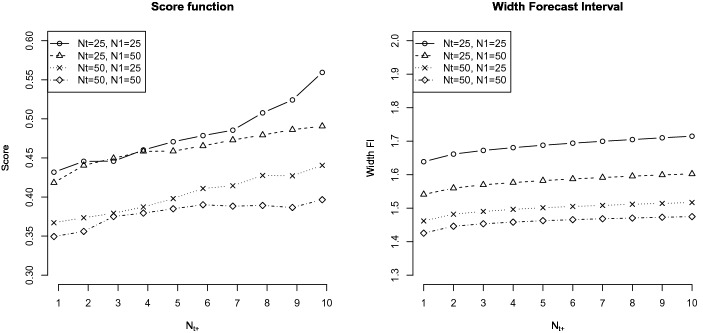
Left: (Quadratic) score function under the different conditions of sample size and time points across the forecast time points. Right: Average width of the forecast intervals (FI) under the different conditions of sample size and time points across the forecast time points.

*Score function* The left-hand panel in Fig. 2 illustrates the (quadratic) score function that indicates the average quadratic difference between the actual factor scores and the forecast factor scores. It provides information about how precise the forecast was under the different conditions and how it changed with more forecast time points. The score function was mostly affected by the number of measurement occasion ($$N_t$$), resulting in similar forecast precision for both sample sizes under the condition of $$N_t=25$$ vs. $$N_t=50$$. Under $$N_t=50$$, the score function resulted in considerably smaller values (for both sample sizes $$N_1=25$$ and $$N_1=50)$$.

*Width of the forecast interval (FI)* The right panel in Fig. 2 shows the average width of the FI across the forecast time points. Across all conditions, the width of the FI increased with additional time points that were forecast (looking like a megaphone). As expected, the FI width decreased with more time points $N_t$ and larger sample sizes $N_1$.

## Results for the Empirical Example

Extended results, detailed parameter estimates and credible intervals for all parameters are given as supplementary online materials. Here, we provide a summary of the main results.

### Overall Model Results

$s=1$ *(no intention to quit) estimates* The baseline scale for *cognitive skills* (IQ; $\varvec{\beta }_{2(s=1)}$) was predictive for all seven within-scales except for the *positive affect* (*no PAP*). Parameter estimates for this construct were negative, that is, persons with higher cognitive skills had lower scores on the within-level scales such as *stress*. The autoregressive coefficients ($\mathbf{B }_{1(s=1)}$) were positive for all seven within-scales under (no intention to quit). This implied that students’ responses on these scales followed a regular pattern. Several *interactions between the within-scales and the cognitive skills* could be observed ($\varvec{\Omega }_{2(s=1)}$).

$s=2$ *(intention to quit) estimates* Persons who switched from the discrete state $s=1$ (no intention to quit) to $s=2$ (intention to quit) showed consistently higher values on all seven scales ($\Delta \varvec{\alpha }_{21(s=2)}$). This provided evidence that the latent states actually indicated attitudes that can be considered to be related to an intention to quit. The autoregressive coefficients were larger under $s=2$ ($\Delta \mathbf{B }_{1(s=2)}$) except for the feeling that students used too much time for studying. This implied that persons who intended to quit had stronger autoregressive coefficients of the affective/cognitive state variables associated with the higher risk of dropout.

*Variance estimates* Estimates of the within-level variances ($\varvec{\sigma }_{\zeta _{1}}^2$) were similar and small for the first five scales (e.g., *content not important* compared to the *no PAP* and *PAN* scales). This implied that time-specific variation was larger for these last two affective scales. The between-level variances ($\varvec{\sigma }_{\zeta _{2}}^2$) were similar across the scales, showing that inter-individual differences were similar across all seven scales. The ICC for the within-level scales lay between 0.496 and 0.692. The forecast will take this aspect into account by using person-specific levels for the individual forecast. The transition probability to return to the state “no intention to drop out” at each time point was estimated at $P_{12}=P(S_{it} = 1| S_{i,t-1} = 2) = 0.097$.

*Markov Switching Model* The results of the parameter estimates of the Markov switching model ($\varvec{\gamma }_{3}$) show that the time-dependent switch was predicted primarily by *PAN* (negative affect) and the scale *being afraid to fail*. This indicates that the switch to an intention of quitting is associated particularly with negative affects and an expectation to fail the final exam. The remaining variables were less predictive for the discrete state change. In addition, the interactions between the cognitive skills (IQ; $\varvec{\gamma }_{4}$) were close to zero.

### Results of the Forecast: All Measurement Occasions

In addition to the 36.1% of persons who had actually quit the studies, a further 37.7% showed a model-based state membership in the latent class $s=2$ (intention to quit) at the final time point $t=50$ (i.e., a total of 73.8% of the students). During the forecast period of five additional time points ($N_{t+}=5$), this percentage increased to 40.2% (i.e., 3 more students were forecast to develop an intention to quit than at $t=50$). The average time point to switch from $s=1$ (no intention to quit) to $s=2$ (intention to quit) was at $t=24.6$ (SD = 8.9). For those persons who later showed an actual dropout, this switch occurred on average at $t=22.2$ (SD = 6.5) which corresponds approximately to the 8th week of the math study program. This was considerably earlier than the actual dropout was observed (on average at $t=45.0$, SD = 11.9) which is approximately the 16th week of the math study program. Note that the period around $t=22.2$ is the critical period when the risk of dropping out of math studies becomes visible and potential interventions should be conducted then at the latest. This corresponds to a difference of 8 weeks before the actual behavior occurs.

**Fig. 3:**
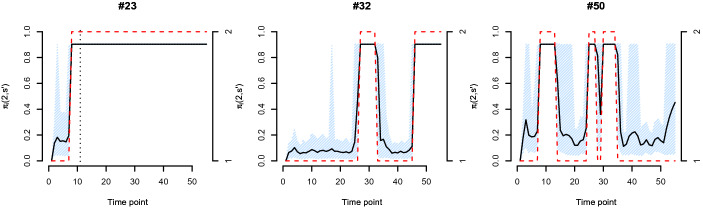
Probabilities for the state switch $\pi _i(2,s')$ for three students. Black indicates the probability with their credible intervals in blue. Red indicates the states $S_{t}$. The left student (#23) actually drops out (indicated with the dotted vertical line). Student # 32 (middle panel) shows an intention to drop out late during the time series, and student #50 (right panel) does not show an intention to drop out during the majority of time points.

Figure 3 illustrates the probabilities $P(S_{t}=2|S_{t-1}=s)$, for example, that is the probability to switch from $S_{it}=1$ to $S_{it}=2$ if a person *i* had no intention to drop out at $t-1$ or to remain in $S_{it}=2$ if the person *i* already showed an intention to drop out at $t-1$. The left student (#23) actually drops out (indicated with the dotted vertical line). Student # 32 (middle panel) shows an intention to drop out late during the time series, and student #50 (right panel) does not show an intention to drop out during the majority of time points.

### Results of the Forecast: Half of the Measurement Occasions

In a second experiment, we used data only from the first $N_t=25$ measurement occasions. We then forecast an additional $N_{t+}=25$ measurement occasions. With this experiment, we wanted to test individual factor scores that were forecast in comparison with the actual factor scores estimated based on all measurement occasions. The forecast of the membership of the discrete latent class at $t=50$ was very similar to the one obtained from the whole sample used above with an overlap of 81% identical classifications in $S_{50}$. The average time point of switching was 23.1 (SD = 10.1); the correlation between the time points from this reduced sample and those estimated from the full sample was 0.62 (see extended results in the online supplementary materials).

## Discussion

In our university dropout study example, we were interested in the separation of stable a priori inter-individual differences (e.g., cognitive abilities), intra-individual changes (e.g., affective/cognitive states) and unobserved heterogeneity (e.g., pre-decisional discrete states of intentions to quit). We were particularly interested in the identification of individuals showing critical states on intra-individual variables or discrete latent states that indicate potential dropout. We used dynamic latent variable modeling (e.g., Asparouhov et al., 2017; 2018) and Bayesian estimation (Gelman et al., 2013), specifically the NDLC-SEM framework (Kelava & Brandt, 2019) which is capable of addressing these different conceptual aspects within one overall model. In a dynamic autoregressive model, we described intra-individual changes of seven within-level latent variables (e.g., subjective importance of content, stress, affective states, etc.). Furthermore, we examined the interaction between these within-level latent state variables and the between-level students’ cognitive abilities variable, in order to explain the transitions to discrete latent states that represent intentions to quit math studies. We were able to show that the development of an intention to drop out is related to baseline and time-specific variables in a complex fashion. We showed that the negative affects expressed in particular are predictive for persons to develop such an intention. In addition, the individuals in these two states (intention/no intention) show a very distinct response pattern on the affective states—they are afraid to fail, show no understanding of the content, believe that they invest to much time in studying, feel stressed and, again, show negative affective states.

Building on previous work of West and Harrison (1997), we presented an adaptation of a Forward Filtering Backward Sampling (FFBS) method that we used for our forecasting problem of time-dependent latent states. The FFBS procedure gives forecasts of both time-dependent continuous latent factors (e.g., affective states) and time-dependent discrete latent states (e.g., intention to quit). We showed that the FFBS is a valid method for forecasting individual trajectories and changes over time. Some initial measures, or a part of a time-series (e.g., the first half of a semester) can be used as a basis to reliably forecast how students will develop with regard to the affective scales. This information is useful to make predictions early on about who might develop an intention to drop out. Our findings are also helpful in indicating when an intervention might be most helpful: The changes in emotional states related to intention to quit occurred, on average, around 8 weeks before any actual dropout, suggesting that early monitoring and early interventions seem more likely to be successful. The results of the simulations study showed that the sensitivity of the FFBS procedure is very good, even for small sample sizes ($N_1 =25$) and a low number of measurements ($N_t =25$). Specificity is acceptable for low sample sizes $N_1=25$, indicating a progressive classification of students at risk (which is acceptable from a substantive point of view). However, specificity improves substantially for sample sizes of $N_1=50$, reaching satisfactory levels.

### Limitations

There are several limitations that need to be reflected on in the context of this paper. First, generally speaking, little is known about the finite sample properties of dynamic latent variable frameworks with respect to the stability of estimates on the different data levels. Systematic simulation studies that examine the balance of the number of individuals ($N_1$) vs. number of measurement occasions ($N_t$) in dynamic models are rare (e.g., Schultzberg, & Muthén, 2018). This has implications for the quality of forecasting in this specific context.

Second, model fit and stability of the model predictions is a critical issue. At this point of research, no global model fit or model fit indices (such as CFI versions for the Bayesian estimator) are available. New research proposed Bayesian adaptations of these fit indices, but they have only been used for very simple CFA models (e.g., Garnier-Villarreal, & Jorgensen, in press). Information criteria that can be used to compare models have also not been systematically investigated for latent variable models with latent dynamic (discrete) states.

Third, test motivation and the willingness to respond to items which are presented repeatedly as part of an ILD assessment are important issues. In our SAM study, we decided to use a small number of items and subsets of scales that were presented three times a week. This had the negative effect of lowering the reliability of the scales which in turn leads to increased variability of the parameter estimates of the effects of constructs of interest. However, we believe that the quality of the data was increased substantially as a result of this decision.

### Future Directions and Open Questions

A number of questions remain open for future research. First, it is unclear how well dynamic SEM will perform compared with statistical learning techniques that allow for classification and prediction of (discrete) states. For example, conditional or temporal restricted Boltzmann machines (e.g., Taylor et al. 2006; Sutskever, & Hinton, 2007) include temporal dependencies. Convolutional layers show promising results in modeling time series by including time-lagged information by one-dimensional convolutions (e.g., convRBM; Lee et al., 2009), as well as recurrent neural networks (RNNs) with long short-term memory cells (LSTM; Hochreiter & Schmidhuber, 1997). However, these methods neither consider different data levels nor they are typically applied to small data sets (e.g., Längkvist et al. 2014). Their suitability for psychological research remains an open question.

Second, since model complexity is important, regularization techniques (both Bayesian and frequentist, e.g., Hastie et al., 2009) will be another aspect that will influence forecasting in models suitable for ILD. Depending on the possibilities to reduce the complexities, alternative procedures might arise which have not yet been extensively discussed in psychometrics. Regularization is important because ILD have substantially lower sample sizes than data that are used in standard machine learning situations.

Third, a combination of techniques both from psychometrics and from machine learning seems to be promising. It will be important to combine their strengths (e.g., the sparsity of psychometric models and their causal orientation with the precision of neural networks). We believe that an important research area will emerge with new perspectives on forecasting in ILD.

## Supplementary Information

Below is the link to the electronic supplementary material.

Supplementary file 1 (pdf 2232 KB)

## Footnotes

1 Note that we use the index *j* as a count index. This means that *j* will be context dependent and redefined at several places in this article.

2 Note that we tested different ARMA-type models. We started with VARMA(1,1) models. The parameters of the MA parts were close to zero. Furthermore, we used shrinkage priors on the VAR coefficients. Particularly, the cross-lagged coefficients were very close to zero. Therefore, for reasons of sparsity and to keep the empirical example simple, we made the simplifying assumption to estimate an AR(1) model as the final model.

3 Note that *j* is a count index. It is used to index elements within a vector. Its length (e.g., $j=1...3$ or $j=1...7$) is redefined in every equation. The count index is used to save space.

4 For a similar definition of strata on potential values within the causality literature, see Frangakis and Rubin (2002).
