# Supplementary material for: Forecasting Intra-individual Changes of Affective States Taking into Account Inter-individual Differences Using Intensive Longitudinal Data from a University Student Dropout Study in Math
Source: Psychometrika. 2022 Apr 2;87(2):533–58. doi: 10.1007/s11336-022-09858-6 (PMC9166886; doi:10.1007/s11336-022-09858-6)
Supplement: Supplementary file 1 — (pdf 2232 KB) [file 11336_2022_9858_MOESM1_ESM.pdf]

## A. Supplementary online material: Reformulation of State-Space representation to DSEM-type representation of the structural model

For a person $i$, let

$$\eta_{1jits} = \mathbf{F}_{jit}\boldsymbol{\theta}_{jts} + v_{jits} \tag{56}$$

with $\mathbf{F}_{jit} := (1, \eta_{1ji,t-1}, \eta_{2i}, \eta_{1ji,t-1}\eta_{2i}, \zeta_{2ji})$, $\boldsymbol{\theta}_{jts} := (\alpha_{21js}, \beta_{1jjs}, \beta_{2js}, \omega_{2jjs}, 1)'$, and $v_{jts} := \zeta_{1jit}$. Note that $\zeta_{1jit}$ has no index $s$, because it is assumed to be class-invariant. Equation (56) is a single row (corresponding to person $i$) from $\boldsymbol{\eta}_{1jts}$ in Equation (26).

Then, some algebra leads to the following expressions:

$$\begin{aligned}
\eta_{1jits} &= (1, \eta_{1ji,t-1}, \eta_{2i}, \eta_{1ji,t-1}\eta_{2i}, \zeta_{2ji})(\alpha_{21js}, \beta_{1jjs}, \beta_{2js}, \omega_{2jjs}, 1)' + \zeta_{1jit} && (57)\\
&= \alpha_{21js} + \beta_{1jjs}\eta_{1ji,t-1} + \beta_{2js}\eta_{2i} + \omega_{2jjs}\eta_{1ji,t-1}\eta_{2i} + \zeta_{2ji} + \zeta_{1jit}\\
&= \alpha_{21js} + \beta_{2js}\eta_{2i} + \zeta_{2ji} + (\beta_{1jjs} + \omega_{2jjs}\eta_{2i})\eta_{1ji,t-1} + \zeta_{1jit}\\
&= \underbrace{\alpha_{1jis}}_{\alpha_{21js}+\beta_{2js}\eta_{2i}+\zeta_{2ji}} + \underbrace{b_{1jjis}}_{\beta_{1jjs}+\omega_{2jjs}\eta_{2i}} \eta_{1ji,t-1} + \zeta_{1jit} && (58)
\end{aligned}$$

Note that $\alpha_{1jis}$ and $b_{1jjis}$ are elements from $\boldsymbol{\alpha}_{1is}$ (see Equation (4)) and $\mathbf{B}_{1is}$ (see Equation (5)) corresponding to the $j$-th within-factor.

Equation (58) corresponds to the $j$-th within-factor (i.e., $j$-th row) in Equation (3).

## B. Supplementary online material: Population definition for the simulation study

Table 2.
Population level parameters: Means and standard deviations (SD) from the empirical example.

| Parameter | Mean | SD |
|---|---|---|
| $\lambda_{10}$ | 1.02 | 0.16 |
| $\lambda_2$ | 0.80 | 0.56 |
| $\beta_{1(s=1)}$ | 0.31 | 0.11 |
| $\beta_{1(s=2)}$ | 0.52 | 0.14 |
| $\beta_{2(s=1)}$ | -0.68 | 0.35 |
| $\beta_{2(s=2)}$ | -0.47 | 0.29 |
| $\alpha_{21(s=1)}$ | 0.02 | 0.12 |
| $\alpha_{21(s=2)}$ | 0.33 | 0.14 |
| $\omega_{2(s=1)}$ | 0.07 | 0.28 |
| $\omega_{2(s=2)}$ | 0.20 | 0.19 |
| $\gamma_1$ | 1.48 | 0.05 |
| $\gamma_2$ | -0.19 | 0.51 |
| $\gamma_3$ | -0.60 | 0.36 |
| $\gamma_4$ | -0.71 | 0.05 |
| $\sigma^2_{\zeta_{1j}}$ | 0.09 | 0.05 |
| $\sigma^2_{\zeta_{2j}}$ | 0.14 | 0.03 |
| $\sigma^2_{\eta_2}$ | 0.20 | 0.05 |
| $\sigma^2_{\epsilon_1}$ | 0.38 | 0.17 |
| $\sigma^2_{\epsilon_2}$ | 0.82 | 0.10 |

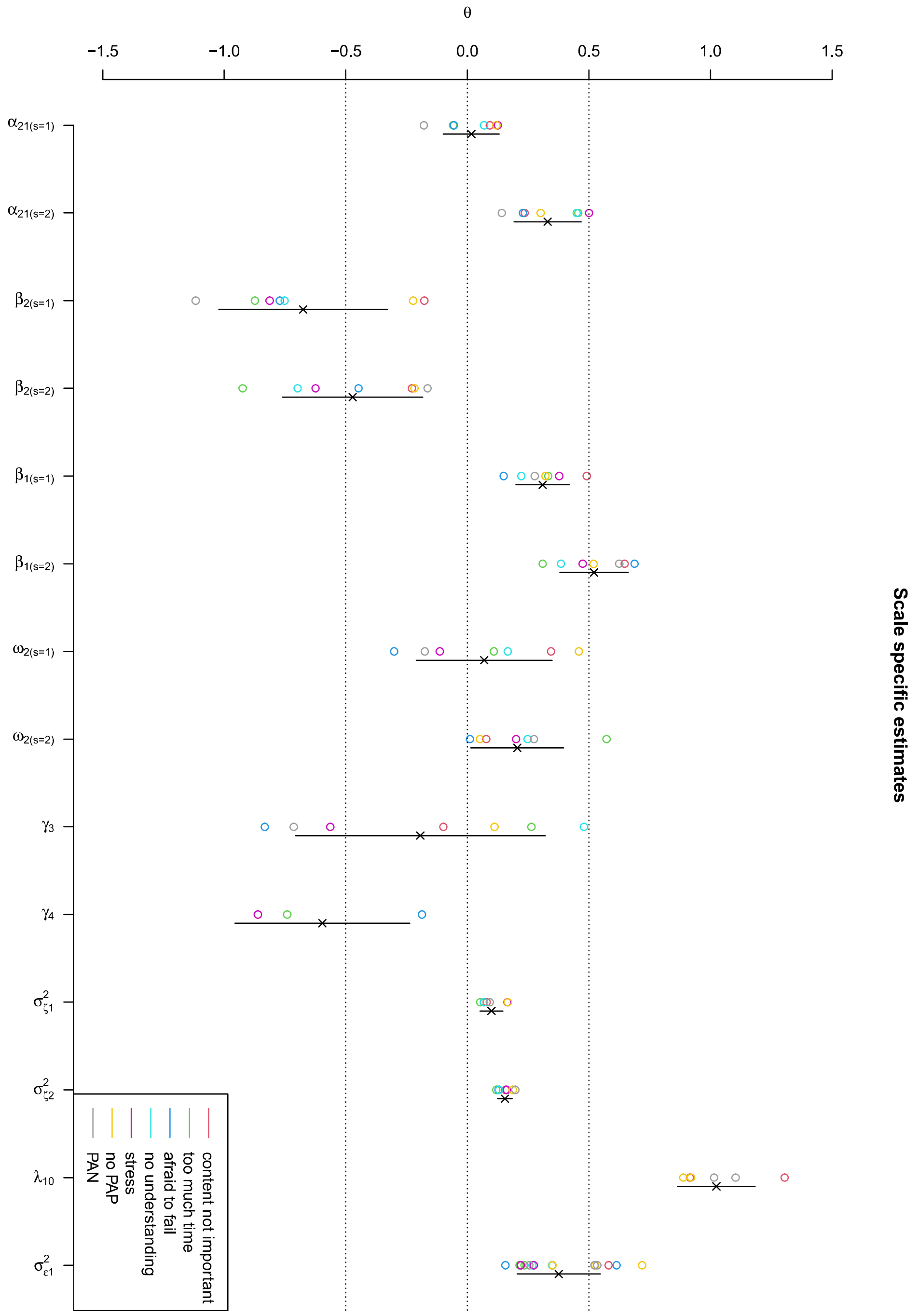


FIGURE 4.
Scale specific parameter estimates from the empirical example. Means and standard deviations for each parameter across scales are indicated with crosses and lines.

## C. Supplementary online material: Parameter estimates from the empirical example

TABLE 3.
Parameter estimates for the latent discrete state $s = 1$ (Mean, SD, 2.5% and 97.5% percentiles of the posterior distribution and the Rhat statistic).

| | Mean | SD | 2.5% | 97.5% | Rhat |
|---|---|---|---|---|---|
| $\alpha_{211(s=1)}$ | 0.09 | 0.03 | 0.02 | 0.16 | 1.00 |
| $\alpha_{212(s=1)}$ | -0.06 | 0.05 | -0.16 | 0.03 | 1.00 |
| $\alpha_{213(s=1)}$ | -0.06 | 0.04 | -0.14 | 0.03 | 1.01 |
| $\alpha_{214(s=1)}$ | 0.07 | 0.05 | -0.02 | 0.16 | 1.02 |
| $\alpha_{215(s=1)}$ | 0.13 | 0.04 | 0.04 | 0.22 | 1.00 |
| $\alpha_{216(s=1)}$ | 0.12 | 0.04 | 0.03 | 0.21 | 1.01 |
| $\alpha_{217(s=1)}$ | -0.18 | 0.06 | -0.29 | -0.06 | 1.02 |
| $\beta_{21(s=1)}$ | -0.18 | 0.09 | -0.35 | -0.01 | 1.00 |
| $\beta_{22(s=1)}$ | -0.87 | 0.15 | -1.18 | -0.60 | 1.00 |
| $\beta_{23(s=1)}$ | -0.77 | 0.12 | -1.02 | -0.56 | 1.03 |
| $\beta_{24(s=1)}$ | -0.75 | 0.14 | -1.02 | -0.49 | 1.01 |
| $\beta_{25(s=1)}$ | -0.81 | 0.13 | -1.09 | -0.57 | 1.01 |
| $\beta_{26(s=1)}$ | -0.22 | 0.12 | -0.46 | 0.00 | 1.01 |
| $\beta_{27(s=1)}$ | -1.12 | 0.17 | -1.45 | -0.79 | 1.01 |
| $\beta_{11(s=1)}$ | 0.49 | 0.04 | 0.40 | 0.57 | 1.07 |
| $\beta_{12(s=1)}$ | 0.33 | 0.04 | 0.25 | 0.41 | 1.02 |
| $\beta_{13(s=1)}$ | 0.15 | 0.04 | 0.06 | 0.24 | 1.07 |
| $\beta_{14(s=1)}$ | 0.22 | 0.05 | 0.12 | 0.32 | 1.02 |
| $\beta_{15(s=1)}$ | 0.38 | 0.04 | 0.30 | 0.45 | 1.01 |
| $\beta_{16(s=1)}$ | 0.32 | 0.05 | 0.22 | 0.41 | 1.01 |
| $\beta_{17(s=1)}$ | 0.28 | 0.04 | 0.20 | 0.35 | 1.01 |
| $\omega_{21(s=1)}$ | 0.34 | 0.11 | 0.15 | 0.58 | 1.12 |
| $\omega_{22(s=1)}$ | 0.11 | 0.09 | -0.07 | 0.29 | 1.01 |
| $\omega_{23(s=1)}$ | -0.30 | 0.10 | -0.50 | -0.11 | 1.04 |
| $\omega_{24(s=1)}$ | 0.17 | 0.10 | -0.03 | 0.37 | 1.01 |
| $\omega_{25(s=1)}$ | -0.11 | 0.07 | -0.27 | 0.03 | 1.00 |
| $\omega_{26(s=1)}$ | 0.46 | 0.13 | 0.21 | 0.72 | 1.01 |
| $\omega_{27(s=1)}$ | -0.18 | 0.08 | -0.34 | -0.02 | 1.05 |

Table 4.
Parameter estimates for the latent discrete state $s = 2$ (Mean, SD, 2.5% and 97.5% percentiles of the posterior distribution and the Rhat statistic).

| | Mean | SD | 2.5% | 97.5% | Rhat |
|---|---|---|---|---|---|
| $\Delta\alpha_{211(s=2)}$ | 0.14 | 0.03 | 0.09 | 0.20 | 1.03 |
| $\Delta\alpha_{212(s=2)}$ | 0.51 | 0.04 | 0.44 | 0.59 | 1.03 |
| $\Delta\alpha_{213(s=2)}$ | 0.28 | 0.03 | 0.23 | 0.34 | 1.01 |
| $\Delta\alpha_{214(s=2)}$ | 0.38 | 0.04 | 0.30 | 0.46 | 1.03 |
| $\Delta\alpha_{215(s=2)}$ | 0.38 | 0.04 | 0.30 | 0.45 | 1.01 |
| $\Delta\alpha_{216(s=2)}$ | 0.18 | 0.04 | 0.11 | 0.26 | 1.03 |
| $\Delta\alpha_{217(s=2)}$ | 0.32 | 0.05 | 0.23 | 0.41 | 1.03 |
| $\Delta\beta_{21(s=2)}$ | -0.05 | 0.08 | -0.22 | 0.11 | 1.05 |
| $\Delta\beta_{22(s=2)}$ | -0.05 | 0.13 | -0.30 | 0.23 | 1.03 |
| $\Delta\beta_{23(s=2)}$ | 0.32 | 0.11 | 0.13 | 0.54 | 1.05 |
| $\Delta\beta_{24(s=2)}$ | 0.05 | 0.13 | -0.20 | 0.31 | 1.07 |
| $\Delta\beta_{25(s=2)}$ | 0.19 | 0.12 | -0.06 | 0.42 | 1.04 |
| $\Delta\beta_{26(s=2)}$ | 0.01 | 0.12 | -0.22 | 0.24 | 1.03 |
| $\Delta\beta_{27(s=2)}$ | 0.95 | 0.15 | 0.68 | 1.25 | 1.04 |
| $\Delta\beta_{11(s=2)}$ | 0.16 | 0.04 | 0.08 | 0.24 | 1.02 |
| $\Delta\beta_{12(s=2)}$ | -0.02 | 0.05 | -0.12 | 0.09 | 1.02 |
| $\Delta\beta_{13(s=2)}$ | 0.54 | 0.05 | 0.45 | 0.62 | 1.11 |
| $\Delta\beta_{14(s=2)}$ | 0.16 | 0.05 | 0.06 | 0.27 | 1.03 |
| $\Delta\beta_{15(s=2)}$ | 0.10 | 0.04 | 0.01 | 0.18 | 1.02 |
| $\Delta\beta_{16(s=2)}$ | 0.20 | 0.05 | 0.10 | 0.30 | 1.02 |
| $\Delta\beta_{17(s=2)}$ | 0.35 | 0.04 | 0.26 | 0.43 | 1.03 |
| $\Delta\omega_{21(s=2)}$ | -0.27 | 0.10 | -0.47 | -0.09 | 1.03 |
| $\Delta\omega_{22(s=2)}$ | 0.46 | 0.13 | 0.22 | 0.73 | 1.02 |
| $\Delta\omega_{23(s=2)}$ | 0.31 | 0.08 | 0.16 | 0.48 | 1.01 |
| $\Delta\omega_{24(s=2)}$ | 0.08 | 0.11 | -0.12 | 0.30 | 1.04 |
| $\Delta\omega_{25(s=2)}$ | 0.31 | 0.10 | 0.13 | 0.51 | 1.00 |
| $\Delta\omega_{26(s=2)}$ | -0.41 | 0.14 | -0.69 | -0.12 | 1.02 |
| $\Delta\omega_{27(s=2)}$ | 0.45 | 0.13 | 0.21 | 0.73 | 1.04 |

| | Mean | SD | 2.5% | 97.5% | Rhat |
|---|---|---|---|---|---|
| $\sigma^2_{\zeta_{11}}$ | 0.06 | 0.00 | 0.05 | 0.06 | 1.00 |
| $\sigma^2_{\zeta_{12}}$ | 0.08 | 0.01 | 0.07 | 0.09 | 1.01 |
| $\sigma^2_{\zeta_{13}}$ | 0.05 | 0.00 | 0.05 | 0.06 | 1.00 |
| $\sigma^2_{\zeta_{14}}$ | 0.07 | 0.01 | 0.06 | 0.08 | 1.00 |
| $\sigma^2_{\zeta_{15}}$ | 0.07 | 0.00 | 0.06 | 0.08 | 1.01 |
| $\sigma^2_{\zeta_{16}}$ | 0.16 | 0.01 | 0.14 | 0.19 | 1.00 |
| $\sigma^2_{\zeta_{17}}$ | 0.17 | 0.01 | 0.15 | 0.19 | 1.02 |
| $\sigma^2_{\zeta_{21}}$ | 0.09 | 0.01 | 0.07 | 0.12 | 1.00 |
| $\sigma^2_{\zeta_{22}}$ | 0.16 | 0.02 | 0.12 | 0.21 | 1.00 |
| $\sigma^2_{\zeta_{23}}$ | 0.12 | 0.02 | 0.09 | 0.16 | 1.00 |
| $\sigma^2_{\zeta_{24}}$ | 0.13 | 0.02 | 0.10 | 0.17 | 1.00 |
| $\sigma^2_{\zeta_{25}}$ | 0.13 | 0.02 | 0.10 | 0.17 | 1.00 |
| $\sigma^2_{\zeta_{26}}$ | 0.16 | 0.02 | 0.12 | 0.21 | 1.00 |
| $\sigma^2_{\zeta_{27}}$ | 0.19 | 0.03 | 0.14 | 0.25 | 1.00 |
| $\sigma^2_{\eta_2}$ | 0.20 | 0.04 | 0.14 | 0.28 | 1.01 |
| $P_{12}$ | 0.10 | 0.00 | 0.09 | 0.10 | 1.00 |

Table 5.
Parameter estimates for the Markov switching model (Mean, SD, 2.5% and 97.5% percentiles of the posterior distribution and the Rhat statistic).

| | Mean | SD | 2.5% | 97.5% | Rhat |
|---|---|---|---|---|---|
| $\gamma_2$ | -1.17 | 0.79 | -2.67 | 0.42 | 1.03 |
| $\gamma_{31}$ | -0.10 | 0.34 | -0.77 | 0.58 | 1.01 |
| $\gamma_{32}$ | 0.26 | 0.33 | -0.40 | 0.91 | 1.02 |
| $\gamma_{33}$ | -0.83 | 0.48 | -1.77 | 0.12 | 1.03 |
| $\gamma_{34}$ | 0.48 | 0.55 | -0.58 | 1.54 | 1.03 |
| $\gamma_{35}$ | -0.56 | 0.42 | -1.41 | 0.23 | 1.03 |
| $\gamma_{36}$ | 0.11 | 0.25 | -0.38 | 0.58 | 1.01 |
| $\gamma_{37}$ | -0.71 | 0.21 | -1.13 | -0.31 | 1.03 |
| $\gamma_{41}$ | -0.74 | 0.68 | -2.10 | 0.54 | 1.06 |
| $\gamma_{42}$ | -0.19 | 0.76 | -1.73 | 1.24 | 1.02 |
| $\gamma_{43}$ | -0.86 | 0.74 | -2.32 | 0.57 | 1.04 |

Table 6.
Parameter estimates for the factor loadings (Mean, SD, 2.5% and 97.5% percentiles of the posterior distribution and the Rhat statistic).

| | Mean | SD | 2.5% | 97.5% | Rhat |
|---|---|---|---|---|---|
| $\lambda_{101}$ | 1.31 | 0.03 | 1.25 | 1.36 | 1.00 |
| $\lambda_{102}$ | 0.92 | 0.03 | 0.86 | 0.97 | 1.00 |
| $\lambda_{103}$ | 0.92 | 0.03 | 0.87 | 0.97 | 1.01 |
| $\lambda_{104}$ | 0.89 | 0.03 | 0.83 | 0.95 | 1.01 |
| $\lambda_{105}$ | 1.10 | 0.02 | 1.06 | 1.14 | 1.00 |
| $\lambda_{106}$ | 1.01 | 0.02 | 0.97 | 1.06 | 1.00 |
| $\lambda_{201}$ | 1.20 | 0.23 | 0.76 | 1.67 | 1.01 |
| $\lambda_{202}$ | 0.41 | 0.22 | 0.04 | 0.87 | 1.01 |

Table 7.
Parameter estimates for the residual variances of the items (Mean, SD, 2.5% and 97.5% percentiles of the posterior distribution and the Rhat statistic).

| | Mean | SD | 2.5% | 97.5% | Rhat |
|---|---|---|---|---|---|
| $\sigma_{\epsilon^2_{11}}$ | 0.35 | 0.01 | 0.32 | 0.37 | 1.00 |
| $\sigma_{\epsilon^2_{12}}$ | 0.22 | 0.01 | 0.20 | 0.24 | 1.00 |
| $\sigma_{\epsilon^2_{13}}$ | 0.58 | 0.02 | 0.55 | 0.62 | 1.00 |
| $\sigma_{\epsilon^2_{14}}$ | 0.23 | 0.01 | 0.22 | 0.25 | 1.00 |
| $\sigma_{\epsilon^2_{15}}$ | 0.21 | 0.01 | 0.20 | 0.23 | 1.00 |
| $\sigma_{\epsilon^2_{16}}$ | 0.16 | 0.01 | 0.14 | 0.18 | 1.02 |
| $\sigma_{\epsilon^2_{17}}$ | 0.61 | 0.02 | 0.57 | 0.66 | 1.01 |
| $\sigma_{\epsilon^2_{18}}$ | 0.27 | 0.01 | 0.25 | 0.29 | 1.00 |
| $\sigma_{\epsilon^2_{19}}$ | 0.35 | 0.01 | 0.32 | 0.38 | 1.00 |
| $\sigma_{\epsilon^2_{110}}$ | 0.27 | 0.01 | 0.25 | 0.30 | 1.00 |
| $\sigma_{\epsilon^2_{111}}$ | 0.22 | 0.01 | 0.20 | 0.24 | 1.00 |
| $\sigma_{\epsilon^2_{112}}$ | 0.35 | 0.02 | 0.32 | 0.39 | 1.01 |
| $\sigma_{\epsilon^2_{113}}$ | 0.53 | 0.02 | 0.49 | 0.57 | 1.00 |
| $\sigma_{\epsilon^2_{114}}$ | 0.72 | 0.03 | 0.67 | 0.77 | 1.00 |
| $\sigma_{\epsilon^2_{115}}$ | 0.52 | 0.02 | 0.49 | 0.56 | 1.00 |
| $\sigma_{\epsilon^2_{116}}$ | 0.26 | 0.01 | 0.23 | 0.28 | 1.00 |
| $\sigma_{\epsilon^2_{117}}$ | 0.53 | 0.02 | 0.50 | 0.57 | 1.00 |
| $\sigma_{\epsilon^2_{21}}$ | 0.82 | 0.10 | 0.64 | 1.04 | 1.00 |
| $\sigma_{\epsilon^2_{22}}$ | 0.73 | 0.09 | 0.56 | 0.93 | 1.00 |
| $\sigma_{\epsilon^2_{23}}$ | 0.92 | 0.11 | 0.72 | 1.16 | 1.00 |

## D. Supplementary online material: Extended results for the empirical example

In this appendix, we present the detailed results for the empirical example predicting student drop out from math. First, we will describe the results for the parameter estimates. Then we will provide information about the forecast.

### *D.1. Overall model results*

Parameter estimates and credible intervals for all parameters are depicted in Figures 5 to 8. Figure 5 shows the results for parameters on the latent discrete state $s = 1$ (no intention to quit). The baseline scale for *cognitive skills* (IQ; indicated by $\boldsymbol{\beta}_{2(s=1)}$) was predictive for all seven within-scales except for the *positive affect* (*no PAP*). Parameter estimates for this construct were negative, that is, persons with higher cognitive skills had lower scores on the within level scales such as *stress*. The auto-regressive coefficients ($\mathbf{B}_{1(s=1)}$) were positive for all seven within-scales under (no intention to quit). This implied that students' responses on these scales followed a regular pattern. Several *interactions between the within-scales and the cognitive skills* could be observed ($\boldsymbol{\Omega}_{2(s=1)}$): For *being afraid to fail* and *negative affect (PAN)* they were negative under $s = 1$, implying that higher *cognitive skills* could diminish the negative effects of these scales. For the scales *content not important* and *no PAP* the interaction effects were positive, implying that persons who simultaneously had higher *cognitive skills* and expressed that they did not find the content important were even more stable on this expression over time.

Figure 6 depicts the differences in the parameters between discrete states $s = 1$ and $s = 2$ (intention to quit). Persons who switched from the discrete state $s = 1$ (no intention to quit) to $s = 2$ (intention to quit) showed consistently higher values on all seven scales ($\Delta\boldsymbol{\alpha}_{21(s=2)}$). This provided evidence that the latent states actually indicated attitudes that can be considered to be related to an intention to quit. The effects of the cognitive skills were similar in state $s = 2$ (intention to quit) compared to $s = 1$ ($\Delta\boldsymbol{\beta}_{2(s=2)}$) except for *PAN* and *being afraid to fail.* For these two scales, the effect of cognitive skills was smaller, that is, persons with different cognitive skills had similar scores on these two scales (e.g. for *PAN* $\beta_{27(s=1)} = -1.117$ under $s = 1$ vs. $\beta_{27(s=2)} = \beta_{27(s=1)} + \Delta\beta_{27(s=2)} = -0.163$ under $s = 2$). The auto-regressive coefficients were larger under $s = 2$ (as indicated with $\Delta\mathbf{B}_{1(s=2)}$) except for the feeling that students used too much time for studying. This implied that persons who intended to quit had stronger auto-regressive coefficients of the dysfunctional affective state variables (e.g. for *afraid to fail*, $\beta_{13(s=1)} = 0.149$ under $s = 1$ vs. $\beta_{13(s=2)} = \beta_{13(s=1)} + \Delta\beta_{13(s=2)} = 0.688$ under $s = 2$). This means that once a student got perturbed away from her baseline affective state, it took longer to return to her baseline value. Under state $s = 2$ (intention to quit) the interactions tended to be smaller in absolute size.

Figure 7 includes the variances at the within and between level as well as the estimate for the transition probability $P_{12} = P(S_{it} = 1|S_{i,t-1} = 2)$. Within-level variances were similar and small for the first five scales (e.g., *content not important* compared to the *no PAP* and *PAN* scales). This implied that time-specific variation was larger for these last two scales. The between-level variances were similar across the scales, showing that inter-individual differences were similar across all seven scales. The ICC for the within-level scales lay between 0.496 and 0.692. This

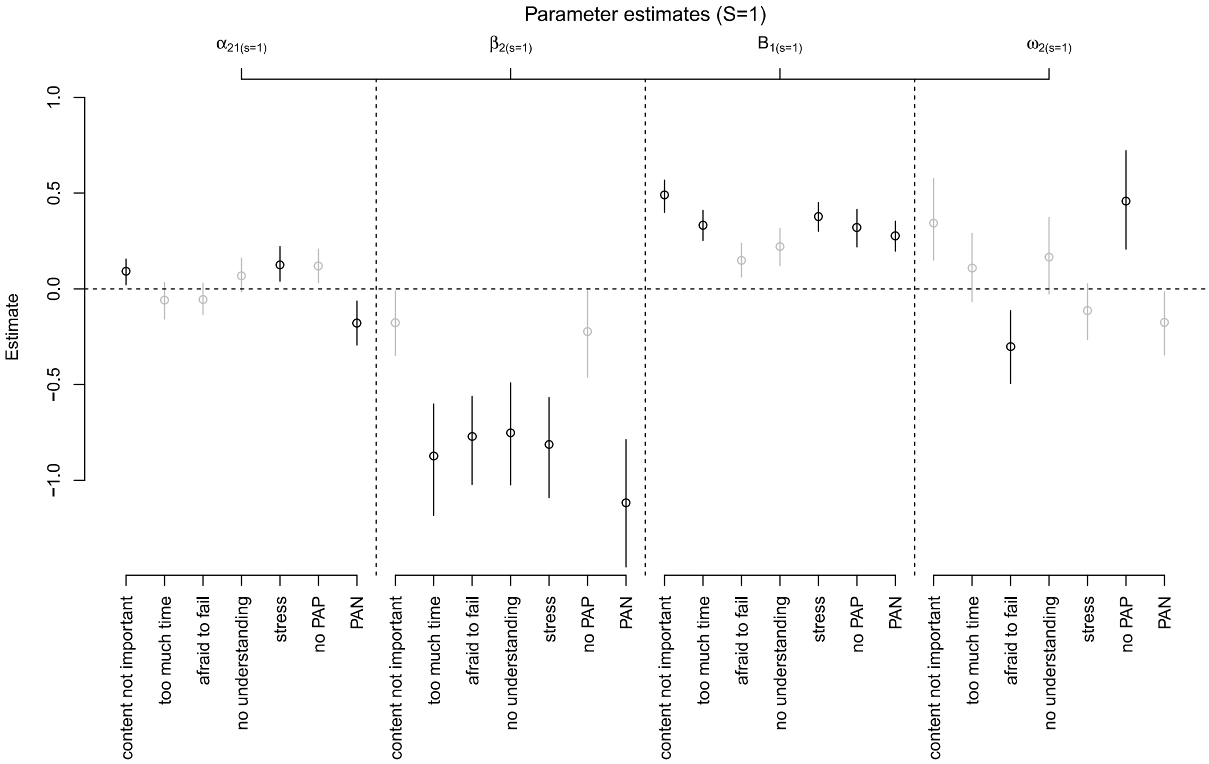


Figure 5.
State-specific parameter estimates from the empirical example for the discrete state $s = 1$ (no intention to quit). Significant estimates (i.e., the 95% credible interval did not include zero) are indicated in black.

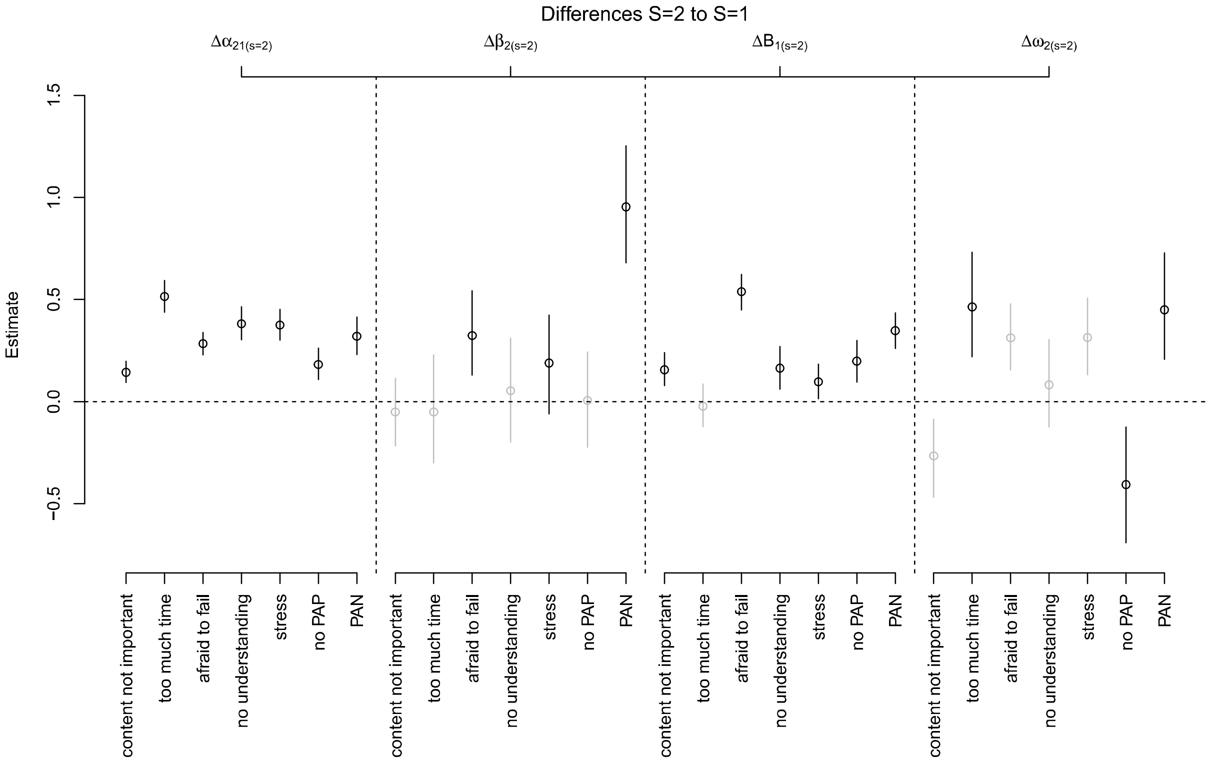


FIGURE 6.
State-specific parameter estimates from the empirical example for the discrete state $s = 2$ (intention to quit). Significant differences between discrete states $s = 1$ and $s = 2$ (i.e., the 95% credible interval did not include zero) are indicated in black.

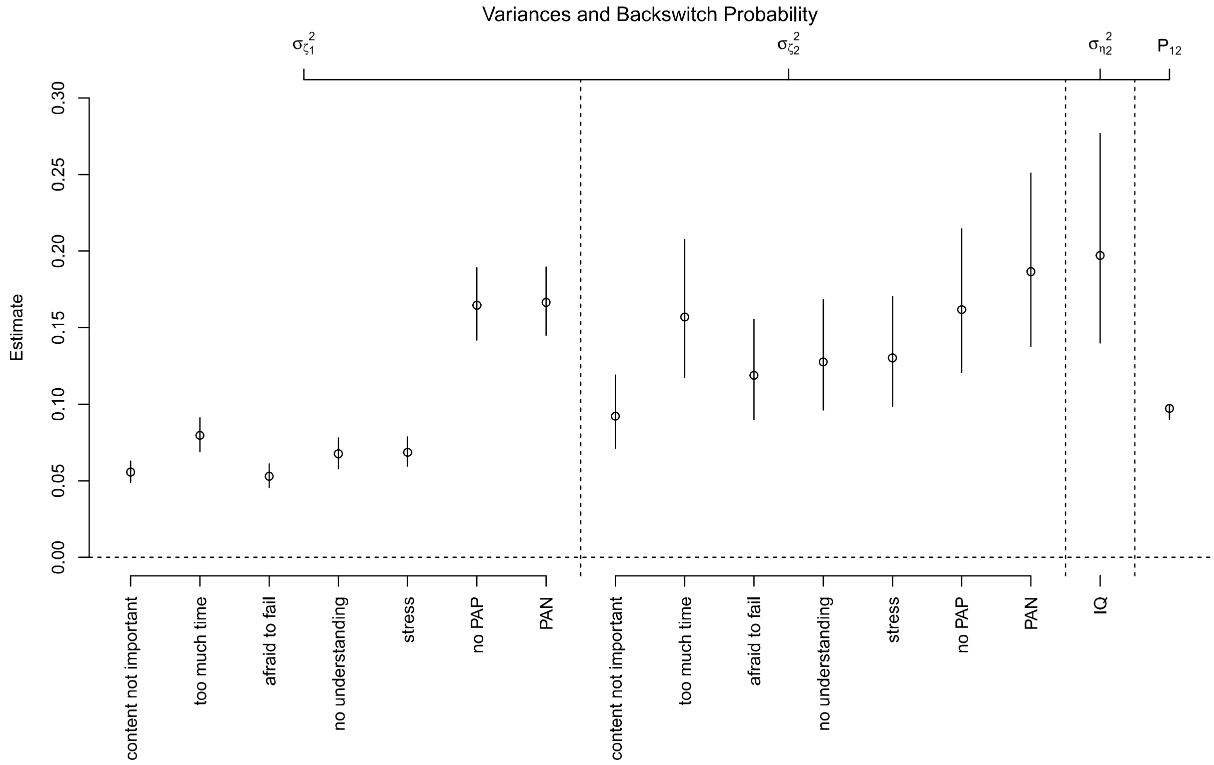


Figure 7.
Parameter estimates from the empirical example for the variances at within and between levels as well as transition probability ($P_{12}$). Significant estimates (i.e., the 95% credible interval did not include zero) are indicated in black.

implied that the scale levels had substantive inter-individual variation. Below, the forecast will take this aspect into account by using person-specific levels (random effects) for the individual forecast. The conditional transition probability from intention to quit to no intention to quit at each time point was estimated at $P_{12} = P(S_{it} = 1|S_{i,t-1} = 2) = 0.097$. Accordingly, the conditional probability to maintain an intention to quit after having this intention at the first time is $P_{22} = P(S_{it} = 2|S_{i,t-1} = 2) = 0.903$. Note that this probability is neither the overall probability for an intention to quit nor the same as the probability of the behavior to actually drop out (which was 36.1%, see below).

Finally, Figure 8 shows the estimates for the prediction of the latent discrete states (Markov switching model). This time-dependent switch was predicted primarily by *PAN* (negative affect) and the scale *being afraid to fail.* This indicates that the switch to an intention of quitting is associated particularly with negative affects and an expectation to fail the final exam. The remaining variables were less predictive for the discrete state change. In addition, the interactions between the cognitive skills (IQ) were close to zero.

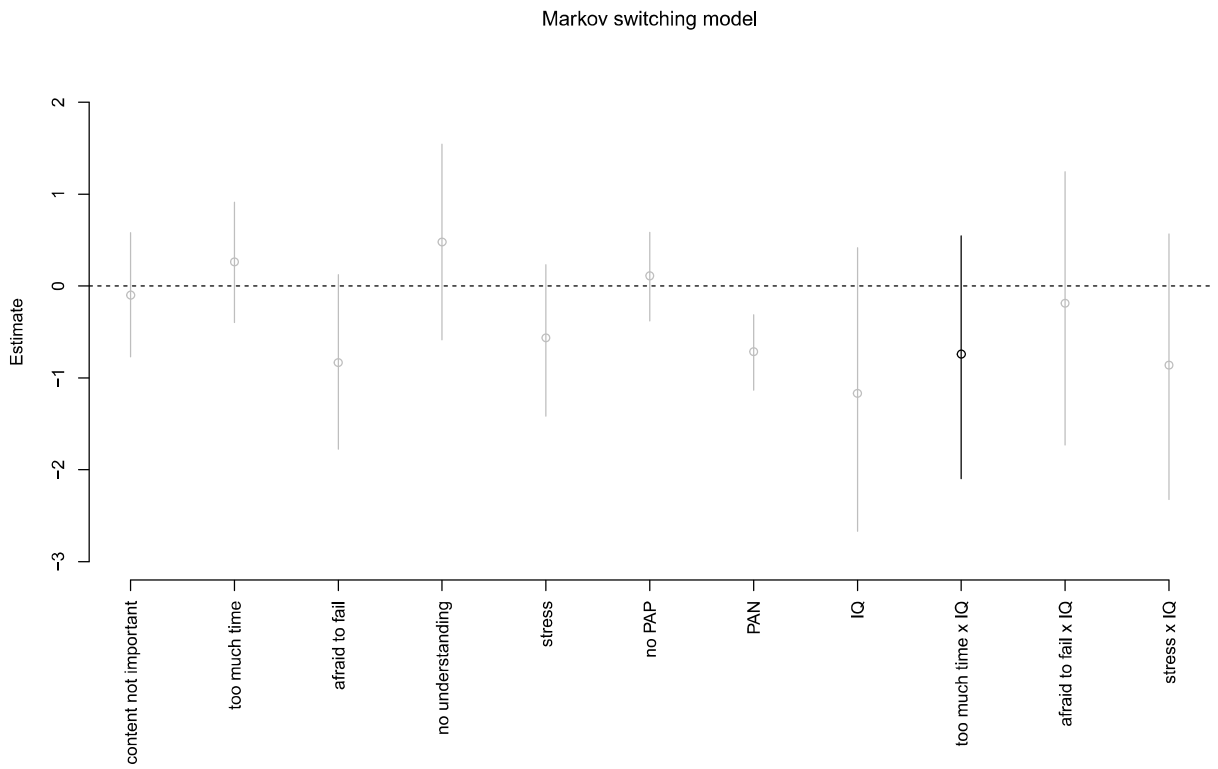


Figure 8.
Parameter estimates from the empirical example for the Markov switching model. Significant estimates (i.e., the 95% credible interval did not include zero) are indicated in black. Negative estimates indicate that persons were more likely to switch to the discrete state $s = 2$ (intention to quit).

*D.2. Results of the forecast: All measurement occasions*

In addition to the 36.1% of persons who had actually quit the studies, a further 37.7% showed a model-based state membership in the latent class $s = 2$ (intention to quit) at the final time point $t = 50$ (i.e., a total of 73.8% of the students). The sensitivity of this intention for the actual observed drop-out was 0.86 and its specificity lay at 0.33. This of course, was expected because we assumed that more persons have an intention to quit than there are actually persons who drop-out (as a behavior).

During the forecast period of 5 additional time points ($N_{t+} = 5$) this percentage increased to 40.2% (i.e., 3 more students were forecast to develop an intention to quit than at $t = 50$). The average time point to switch from $s = 1$ (no intention to quit) to $s = 2$ (intention to quit) was at $t = 24.6$ ($SD = 8.9$). For those persons who later showed an actual drop-out, this switch occurred on average at $t = 22.2$ ($SD = 6.5$) which corresponds approximately to the 8th week of the math study program. This was considerably earlier than the actual drop-out was observed (on average at $t = 45.0$, $SD = 11.9$) which is approximately the 16th week of the math study program. Note that the period around $t = 22.2$ is the critical period when the risk of dropping out of math studies becomes visible and potential interventions should be conducted then at the latest. This corresponds to a difference of 8 weeks before the actual behavior occurs.

Figure 9 shows the average trends of the seven within scales by the two discrete states $s = 1$ (no intention to quit) and $s = 2$ (intention to quit) separately. These scores were sampled from the posterior distribution using the estimated factor scores during time points 1 through $N_t = 50$ and the forecast factor scores for the additional 5 time points ($N_{t+} = 5$). The number of persons used to average these factor scores were different at each time point (estimates for class-membership were also drawn from the posterior distribution). Persons with an intention to quit ($s = 2$) exhibited higher average scores particularly in the scales "too much time", "afraid to fail", "no understanding", "stress" and "PAN". The scores for "no PAP" were overlapping and the scores for the scale "content not important" were slightly higher for persons with an intention to quit.

The top panels (first row) in Figure 10 illustrate individual trajectories of two randomly selected students. The smooth red lines show how the FFBS algorithm recovers the actual trajectories based on the factor scores (blue). For the additional time points beyond $N_t = 50$, the forecast illustrates the future development of the students. Note how student #2 (first column) in particular shows different trajectories compared to student student #5 in the two scale "afraid to fail" and "PAN" which are indicative for their switch to the discrete state $s = 2$.

Figure 11 illustrates the probabilities $\pi_i(2, s')$, that is the probability to switch from $s = 1$ to $s = 2$ if a person had no intention to drop out at $t - 1$ or to remain in $s = 2$ if the person already showed an intention to drop out at $t - 1$. The left student (#23) actually drops out (indicated with the dotted vertical line). Student # 32 (middle panel) shows an intention to drop out late during the time series, and student #50 (right panel) does not show an intention to drop out during the majority of time points.

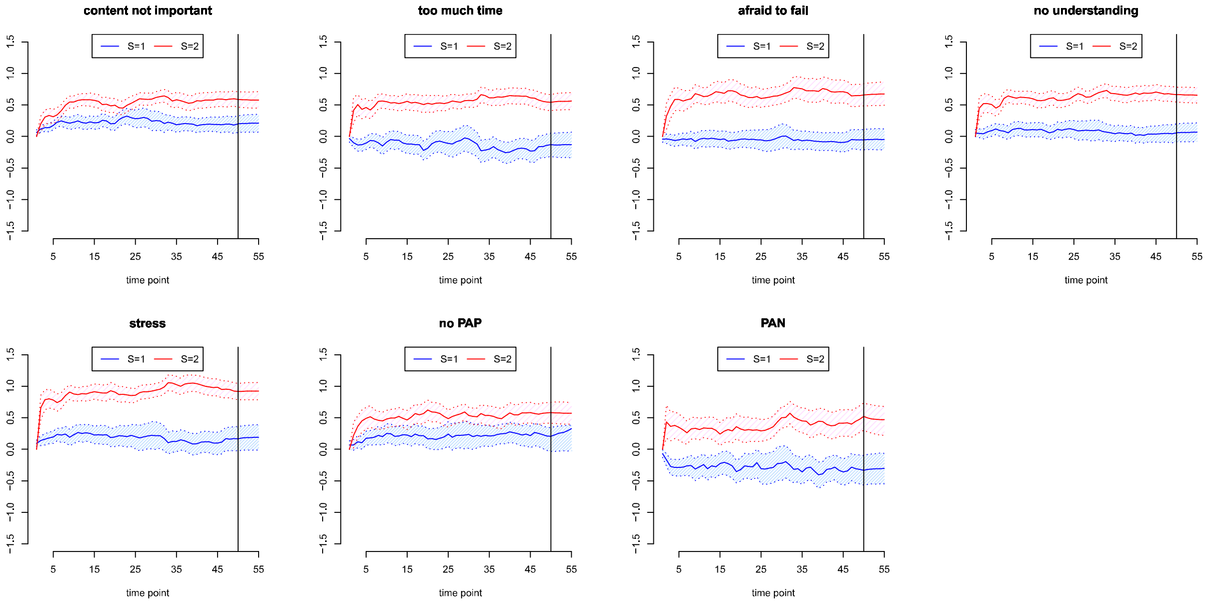


FIGURE 9.
State-specific average trajectories and their forecast beyond $N_t = 50$. $s = 1$ (no intention to quit) and $s = 2$ (intention to quit). Averages were taken at each time point based on the estimated membership in the discrete latent classes.

### *D.3. Results of the forecast: Half of the measurement occasions*

In a second experiment, we used data only from the first $N_t = 25$ measurement occasions. We then forecast an additional $N_{t+} = 25$ measurement occasions. With this experiment, we wanted to test individual factor scores that were forecast in comparison to the actual factor scores estimated based on all measurement occasions. The forecast of the membership of the discrete latent class at $t = 50$ was very similar to the one obtained from the whole sample used above with an overlap of 81% identical classifications in $S_{50}$. The average time point of switching was 23.1 ($SD = 10.1$); the correlation between the time points from this reduced sample and those estimated from the full sample was 0.62.

The bottom panels (second row) in Figure 10 illustrate this forecast for two selected scales for same two students as above. The factor score estimates obtained from the original complete sample (with all measurement occasions) are shown in blue. They are used as a reference to judge the forecast (red). The forecast intervals (FI) include these factor scores reliably. As is typical for the FFBS method, the method is used as forecast for data points $N_{t+}$, and it smoothes over the actual data for the initial time points $1, \ldots N_t$ as can be seen nicely in Figure 10 (see also West & Harrison, 1997; Petris et al., 2009). Thus, the estimates between $t = 1, \ldots 25$ are smoothed predictions of the actual factor scores. Note that the prediction takes the inter-individual different levels directly into account using the random effect estimates.

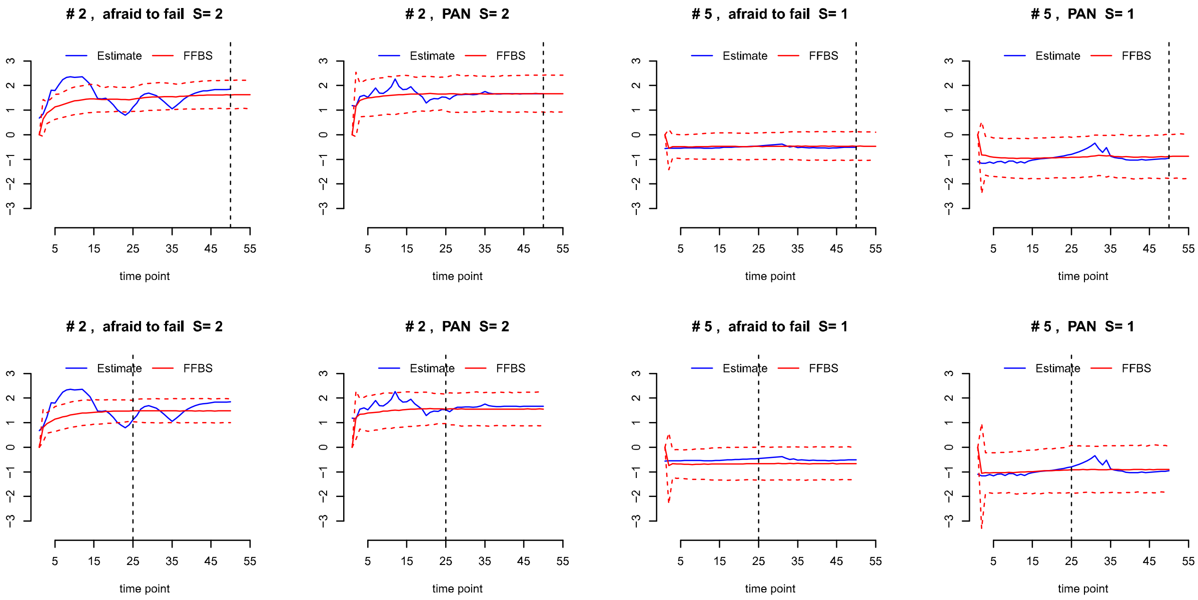


Figure 10.
Individual trajectories for two students (#2, #5). Blue indicates the factor score estimate based on all available measurement occasions. Red indicates the forecast ($t > 50$) and the smoothing ($t < 50$) in the top panels and respective lines for a subset of the data with only 25 measurement occasions in the bottom panels. $S$ indicates the final membership in the discrete state (intention to quit) at $t = 55$ (top panels) or $t = 50$ (bottom panels).

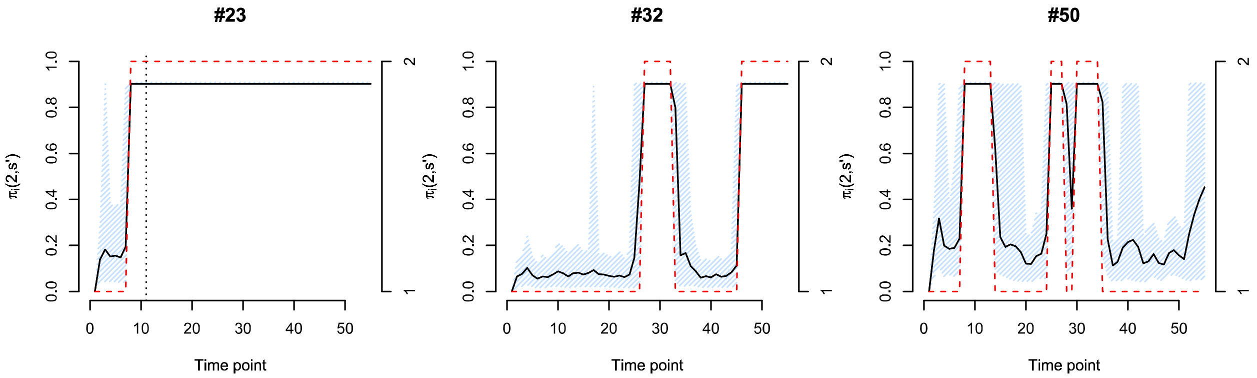


Figure 11.
Probabilities for the state switch $\pi_i(2, s')$ for three students. Black indicates the probability with their credible intervals in blue. Red indicates the states $S$. The left student (#23) actually drops out (indicated with the dotted vertical line). Student # 32 (middle panel) shows an intention to drop out late during the time series, and student #50 (right panel) does not show an intention to drop out during the majority of time points.
